# Innovative myeloid ecotropic viral integration site 1‐engineered exosome platform combating oxidative stress and inflammation in acute pulmonary damage

**DOI:** 10.1002/btm2.70150

**Published:** 2026-05-14

**Authors:** Yukun Zhang, Ke Pang, Zefa Ling, Shouyong Wang, Bo Hu, Surong Deng, Zhao Yang

**Affiliations:** ^1^ Department of Respiratory and Critical Care Medicine The Affiliated Yongchuan Hospital of Chongqing Medical University Chongqing China; ^2^ Department of General Ward The Affiliated Yongchuan Hospital of Chongqing Medical University Chongqing China; ^3^ Department of Neurology The Affiliated Yongchuan Hospital of Chongqing Medical University Chongqing China; ^4^ Department of Anaesthesiology The Affiliated Yongchuan Hospital of Chongqing Medical University Chongqing China; ^5^ Department of Blood Transfusion The Affiliated Yongchuan Hospital of Chongqing Medical University Chongqing China

**Keywords:** acute lung injury, curcumin, exosome engineering, Meis1, oxidative stress

## Abstract

Phosgene‐induced acute lung injury (P‐ALI) remains a severe clinical challenge due to the absence of targeted therapies. In this study, we developed a novel therapeutic strategy based on myeloid ecotropic viral integration site 1‐engineered exosomes loaded with curcumin (Meis1‐Exo@Cur). This nanotherapeutic platform exhibited potent protective effects in both cellular and animal models of P‐ALI, significantly reducing inflammation, oxidative stress, and apoptosis while promoting tissue repair. Mechanistically, Meis1‐Exo@Cur exerted its therapeutic effects through coordinated inhibition of the nuclear factor kappa‐B/mitogen‐activated protein kinase signaling pathways and activation of the Nrf2/HO‐1 pathway, accompanied by remodeling of the immune microenvironment. These findings identify Meis1‐Exo@Cur as a promising therapeutic candidate for P‐ALI and provide a potential framework for exosome‐based precision therapy in inflammatory lung diseases.

## Graphical abstract

Schematic illustration of the molecular mechanism by which myeloid ecotropic viral integration site 1‐engineered exosomes loaded with curcumin (Meis1‐Exo@Cur) enhances tissue regeneration and repair in phosgene‐induced acute lung injury (P‐ALI) (Created in BioRender).

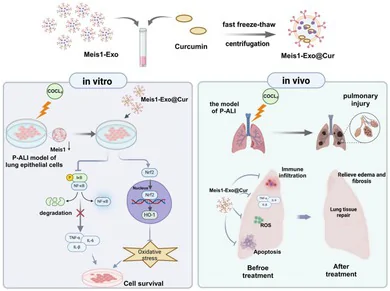

**Translational Impact Statement**

The absence of targeted therapies for phosgene‐induced acute lung injury (P‐ALI) has created a critical bottleneck in treating this severe clinical condition. Here, we present an innovative therapeutic strategy based on myeloid ecotropic viral integration site 1‐engineered exosomes loaded with curcumin (Meis1‐Exo@Cur). This nanotherapeutic platform demonstrated potent protective effects in both cellular and animal models of P‐ALI, significantly reducing inflammation, oxidative stress, and apoptosis while promoting tissue repair. Mechanistically, Meis1‐Exo@Cur exerts its therapeutic effects through coordinated inhibition of the nuclear factor kappa‐B/mitogen‐activated protein kinase signaling pathways and activation of the Nrf2/HO‐1 pathway, accompanied by remodeling of the immune microenvironment. By bridging the translational gap between mechanistic insights and clinical application, this platform establishes Meis1‐Exo@Cur as a promising therapeutic candidate for P‐ALI while providing a potential framework for exosome‐based precision therapy in inflammatory lung diseases.

**Abbreviations**
ALI: acute lung injury
ALT: alanine aminotransferase
ANOVA: analysis of variance
AST: aspartate aminotransferase
BALF: bronchoalveolar lavage fluid
CCK‐8: Cell Counting Kit‐8
CK: creatine kinase
CREA: creatinine
Cur: curcumin
DEG: differentially expressed gene
DEP: differentially expressed protein
DLC: drug loading content
DLE: drug loading efficiency
ELISA: enzyme‐linked immunosorbent assays
Exo: exosome
GO: Gene Ontology
H&E: hematoxylin and eosin
LASSO: Least Absolute Shrinkage and Selection Operator
LDH: lactate dehydrogenase
LPS: lipopolysaccharide
Meis1: myeloid ecotropic viral integration site 1
Meis1‐Exo@Cur: meis1‐engineered mesenchymal stem cell‐derived exosomes Loaded with curcumin
Meis1‐Exos: meis1‐engineered mesenchymal stem cell‐derived exosomes
MSC: mesenchymal stem cell
NTA: nanoparticle tracking analysis
P‐ALI: phosgene‐induced acute lung injury
PI: propidium iodide
PPI: protein–protein interaction
*P*–*V*
: pressure–volume
RNA‐seq: high‐throughput RNA sequencing
ROS: reactive oxygen species
RV: residual volume
SD: standard deviation
TALE: three amino acid loop extension
TLC: total lung capacity
Treg: regulatory T cell
W/D: wet‐to‐dry

## 1 INTRODUCTION

Acute lung injury (ALI) is a common and life‐threatening clinical syndrome characterized by disruption of the alveolar epithelial and capillary endothelial barriers, excessive inflammatory responses, and impaired gas exchange. It progresses rapidly and is associated with high mortality rates.^1^, ^2^, ^3^ Phosgene, a highly toxic inhalational agent, is a major cause of ALI, particularly in chemical accidents and warfare exposure. Phosgene inhalation can lead to alveolar epithelial cell necrosis, pulmonary edema, and diffuse alveolar damage.^4^ Currently, treatment for phosgene‐induced ALI (P‐ALI) primarily depends on supportive care, including mechanical ventilation, fluid management, and anti‐inflammatory medications. However, effective targeted interventions addressing the underlying pathological mechanisms remain lacking.^5^, ^6^ Given the acute onset, severe progression, and often irreversible tissue damage caused by phosgene exposure, there is an urgent need to develop novel therapeutic strategies with integrated anti‐inflammatory, antioxidant, and tissue‐repair functions.^7^, ^8^

In recent years, mesenchymal stem cells (MSCs) have attracted considerable attention for their regenerative and immunomodulatory effects in the treatment of ALI.^9^, ^10^ However, increasing evidence suggests that the therapeutic effects of MSCs are largely mediated through their secreted exosomes (Exos) rather than direct cell replacement.^11^, ^12^ Exos, as nanoscale intercellular communication vesicles, are rich in bioactive molecules and play essential roles in modulating inflammation, oxidative stress, and apoptosis, while also exhibiting low immunogenicity and high biocompatibility.^13^, ^14^ Furthermore, mesenchymal stem cell‐derived exosomes (MSC‐Exos) have demonstrated significant potential for targeted delivery in various disease models due to their innate tissue‐homing ability and tropism toward injured sites.^15^ Studies have suggested that adhesion molecules and chemokine receptors on the surface of MSC‐Exos, including CXCR4 and integrins, may facilitate their preferential accumulation at sites of inflammation or injury, especially in lung tissue.^16^, ^17^, ^18^ Therefore, this study selected MSC‐derived Exos as the delivery vehicle for curcumin (Cur) not only for their excellent biocompatibility and low immunogenicity but also for their potential lung‐targeting capability, which holds promise for achieving efficient drug accumulation in injured lung tissues.^19^, ^20^ Moreover, engineering Exos and loading them with functional therapeutics represents an effective strategy to further improve their efficacy, offering a promising platform for coordinated multitarget intervention in complex and rapidly progressing diseases such as ALI.^21^, ^22^

Myeloid ecotropic viral integration site 1 (Meis1), a transcription factor of the three amino acid loop extension (TALE) family, plays important roles in stem cell maintenance, organ development, and stress responses.^23^ In the present study, integrated analysis of high‐throughput RNA sequencing (RNA‐seq), machine learning algorithms, and protein–protein interaction (PPI) networks revealed that Meis1 was significantly downregulated in P‐ALI, suggesting that it may serve as a key regulatory gene in the injury response. Cur, a natural polyphenolic compound derived from turmeric, exhibits diverse pharmacological activities, including anti‐inflammatory, antioxidant, antifibrotic, and immunomodulatory effects, and has been widely studied in inflammatory diseases.^24^, ^25^ However, its clinical application remains limited by poor water solubility, chemical instability, and low in vivo bioavailability.^26^ Engineering Exos to encapsulate Cur represents a promising strategy to improve its stability and targeting efficiency, while also offering a potential platform for synergistic drug and gene delivery in lung disease treatment.

Given the involvement of multiple signaling pathways and dynamic immune cell infiltration in P‐ALI, single‐omics approaches are insufficient to comprehensively elucidate therapeutic mechanisms. Therefore, this study employed an integrated multi‐omics strategy to systematically investigate the molecular networks and signaling pathways regulated by Meis1‐engineered MSC‐derived Exos loaded with Cur (Meis1‐Exo@Cur) during ALI repair. Multi‐omics integration has attracted increasing attention in inflammatory and injury‐related diseases because it enables the simultaneous capture of dynamic transcriptional changes and protein‐level regulatory events.^27^, ^28^, ^29^ In this study, differentially expressed genes (DEGs) and differentially expressed proteins (DEPs) were jointly analyzed in combination with immune cell infiltration profiling and pathway enrichment analysis to identify the key molecular targets and signaling cascades underlying the anti‐inflammatory, antioxidant, and anti‐apoptotic effects of Meis1‐Exo@Cur. This systematic analysis not only advances the mechanistic understanding of P‐ALI but also provides a basis for the discovery of potential therapeutic targets and biomarkers.

To systematically investigate the functional role and therapeutic potential of Meis1 in P‐ALI, this study developed an engineered exosome‐based delivery system loaded with Cur (Meis1‐Exo@Cur), with Meis1 serving as the core regulatory element. This platform was designed to combine gene regulation with pharmacological intervention to achieve synergistic anti‐inflammatory, antioxidant, and tissue‐repair effects. To further elucidate the underlying mechanisms, an integrated multi‐omics strategy incorporating transcriptomic and proteomic profiling was employed to comprehensively characterize signaling pathway modulation and immune microenvironment remodeling during lung injury repair.

In summary, this study proposes a novel therapeutic strategy by constructing a “Meis1‐engineered exosome + Curcumin” delivery platform and systematically explores its regulatory mechanisms and therapeutic efficacy in a P‐ALI model through multi‐omics data mining. This approach may not only overcome the intrinsic limitations of Cur in vivo but also offer a mechanistically informed and potentially translatable strategy for targeted ALI treatment.

## 2 MATERIALS AND METHODS

### 2.1 Ethics Statement

All animal experiments were conducted in accordance with relevant ethical guidelines and regulations for the care and use of laboratory animals, including the Animal Research: Reporting of In Vivo Experiments (ARRIVE) guidelines, and were approved by the Institutional Animal Care and Use Committee (IACUC). A total of 48 healthy adult male C57BL/6 mice were included in this study, of which 36 were used for the primary evaluation of therapeutic efficacy and safety and 12 for the in vivo biodistribution study. Randomization and blinding were applied throughout the experimental design to reduce bias. All mice were maintained under humane housing and handling conditions, and every effort was made to minimize animal suffering. At the end of the experiments, all animals were euthanized humanely under ether anesthesia.

For further details, please refer to Supporting Information S1: Appendix.

## 3 RESULTS

### 3.1 Transcriptomic analysis reveals downregulation of Meis1 in the progression of P‐ALI

To identify key regulatory factors involved in P‐ALI, we established a P‐ALI mouse model by exposing mice to phosgene gas (8.33 mg/m^3^) for 10 min in an acrylic exposure chamber, followed by transcriptomic profiling of lung tissues from the Control and P‐ALI groups using RNA‐seq (Figure 1a). Compared with the Control group, mice in the P‐ALI group exhibited significantly decreased tidal volume and minute ventilation, accompanied by a marked increase in respiratory rate (Figure S2A). FlexiVent analysis further showed significantly increased dynamic lung resistance and decreased lung compliance, indicating elevated airway resistance and impaired lung elasticity (Figure S2B). Pressure–volume (*P*–*V*) curve analysis revealed a significant increase in residual volume (RV) and a decrease in total lung capacity (TLC), suggesting impaired pulmonary ventilation (Figure S2C). Hematoxylin and eosin (H&E) staining showed prominent inflammatory cell infiltration and structural disruption in alveolar architecture in the P‐ALI group (Figure S2D). In addition, the lung wet‐to‐dry (W/D) ratio was significantly elevated, indicating aggravated pulmonary edema (Figure S2E), whereas terminal deoxynucleotidyl transferase deoxyuridine triphosphate (dUTP) nick end labeling (TUNEL) staining revealed a marked increase in apoptotic cells in lung tissues from P‐ALI mice (Figure S2F). These findings confirmed the successful establishment of the P‐ALI mouse model.

**FIGURE 1:**
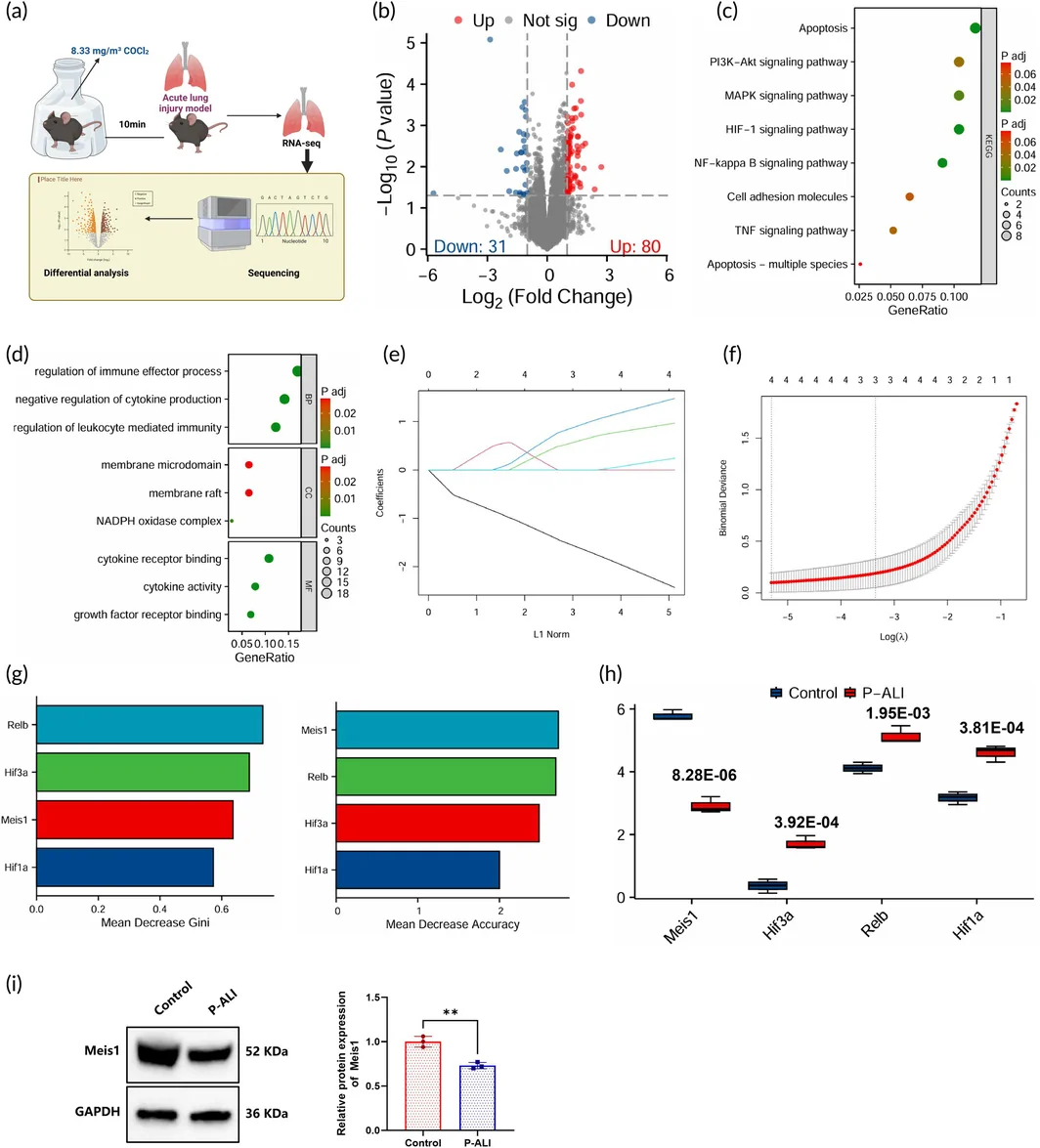
Identification and functional enrichment analysis of differentially expressed genes (DEGs) in phosgene‐induced acute lung injury (P‐ALI). (a) Workflow of bioinformatic analysis (Created in BioRender); (b) volcano plot of DEGs identified by high‐throughput RNA sequencing (RNA‐seq) in lung tissues from P‐ALI and Control groups; (c) Kyoto Encyclopedia of Genes and Genomes pathway enrichment analysis showing involvement of DEGs in key signaling pathways; (d) Gene Ontology enrichment analysis illustrating the biological processes associated with DEGs; (e) Least Absolute Shrinkage and Selection Operator (LASSO) coefficient profiles of DEGs; (f) selection of the optimal lambda value in the LASSO model; (g) variable importance plots from random forest analysis, where higher Mean Decrease Gini and Mean Decrease Accuracy values indicate greater gene importance; (h) Box plots showing differential expression of four feature genes. Each group included three animals; (i) Western blot analysis of myeloid ecotropic viral integration site 1 (Meis1) protein expression levels in lung tissues from Control and P‐ALI mice, along with quantitative statistical analysis. **p < 0.01. HIF‐1, hypoxia‐inducible factor‐1; MAPK, mitogen‐activated protein kinase; NF‐κB, nuclear factor kappa‐B; PI3K‐Akt, phosphatidylinositol 3‐kinase/protein kinase B. GAPDH: glyceraldehyde‐3‐phosphate dehydrogenase.

RNA‐seq analysis revealed 111 DEGs between the P‐ALI and Control groups, including 80 upregulated and 31 downregulated genes (Figure 1b). Kyoto Encyclopedia of Genes and Genomes (KEGG) enrichment analysis showed that these DEGs were mainly involved in apoptosis, phosphatidylinositol 3‐kinase/protein kinase B (PI3K‐Akt), mitogen‐activated protein kinase (MAPK), hypoxia‐inducible factor‐1 (HIF‐1), and nuclear factor kappa‐B (NF‐κB) signaling pathways (Figure 1c). Gene Ontology (GO) analysis further revealed significant enrichment in immune effector processes, membrane microdomains, Nicotinamide Adenine Dinucleotide Phosphate (NADPH) oxidase complex, and cytokine receptor binding (Figure 1d).

To further identify key feature genes, regression analysis was performed on the 111 DEGs to remove redundant genes with similar characteristics. Model performance was assessed by plotting the model object and performing cross‐validation with the cv.glmnet function to determine the optimal lambda value (Figure 1e). Genes with non‐zero coefficients under the optimal lambda were defined as disease‐associated feature genes, yielding four candidates: Meis1, Hif1a, Hif3a, and Relb (Figure 1f). Random forest analysis showed that all four genes contributed substantially to disease classification. Relb ranked highest based on Mean Decrease Gini, whereas Meis1 and Relb showed the greatest importance according to Mean Decrease Accuracy (Figure 1g). Box plot analysis further demonstrated that Meis1 was significantly downregulated in the P‐ALI group, whereas Hif1a, Hif3a, and Relb were significantly upregulated. Among them, Meis1 exhibited the most prominent differential expression pattern (Figure 1h). To validate these findings at the protein level, western blot (WB) analysis was performed using lung tissues from Control and P‐ALI mice. Consistent with the transcriptomic results, Meis1 protein expression was markedly decreased in the P‐ALI group (Figure 1i), confirming that Meis1 was suppressed during the progression of P‐ALI.

These bioinformatic and experimental findings indicate that Meis1 is significantly downregulated in P‐ALI lung tissues. To our knowledge, neither the expression pattern nor the functional role of Meis1 in P‐ALI has been previously reported. This finding provides new insight into the molecular basis of phosgene‐induced pulmonary injury and suggests that Meis1 may represent a potential therapeutic target in this condition.

### 3.2 Meis1 overexpression alleviates oxidative stress and apoptosis in P‐ALI

To validate the transcriptomic findings regarding Meis1 expression in mouse alveolar epithelial cells, an in vitro P‐ALI model was established using the MLE15 cell line (species: *Mus musculus*; sex: male; tissue of origin: lung alveolar epithelium; official cell line name: MLE‐15; (Research Resource Identifier (RRID): CVCL_0459; United States; catalog number: CSC‐I9200L) (Figure 2a). Flow cytometry and Cell Counting Kit‐8 (CCK‐8) assays showed that, compared with the Control group, the P‐ALI group exhibited significantly reduced cell viability and increased apoptosis (Figure S2A,B). Consistently, WB analysis revealed marked upregulation of the pro‐apoptotic proteins cleaved caspase‐3 and Bax, together with downregulation of the anti‐apoptotic protein Bcl2 in the P‐ALI group (Figure S2C). Oxidative stress assessment further showed significantly elevated intracellular reactive oxygen species (ROS) levels and lactate dehydrogenase (LDH) release in P‐ALI cells (Figure S2D,E). These findings confirmed the successful establishment of the in vitro P‐ALI model. Both reverse transcription‐quantitative polymerase chain reaction (RT‐qPCR) and WB analyses demonstrated that Meis1 mRNA and protein levels were significantly decreased in the P‐ALI group compared with the Control group (Figure 2b,c). Given the reported role of Meis1 in promoting stem cell self‐renewal, proliferation, and differentiation,^30^ Meis1 was hypothesized to act as a key regulator in the repair of alveolar epithelial injury during P‐ALI.

**FIGURE 2:**
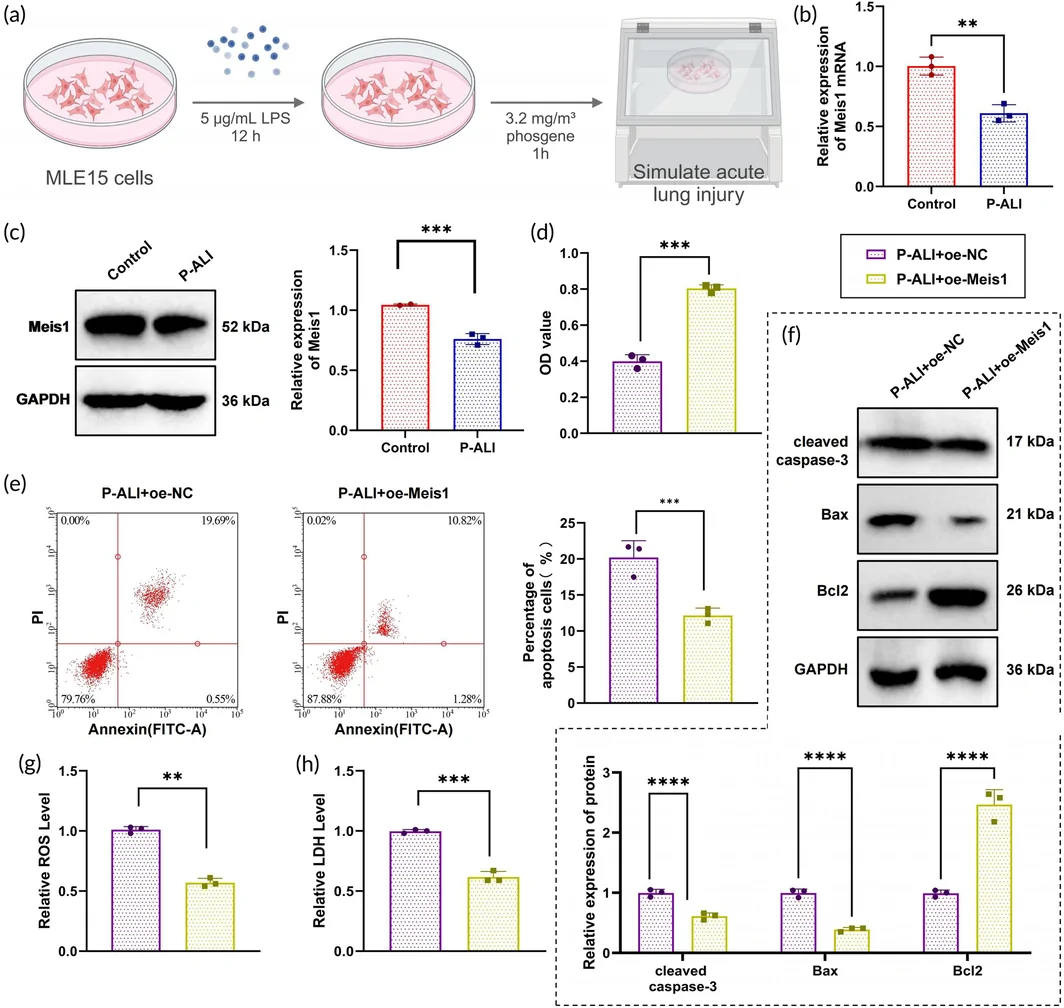
Expression and protective role of myeloid ecotropic viral integration site 1 (Meis1) in phosgene‐induced acute lung injury (P‐ALI). (a) Schematic illustration of the in vitro P‐ALI model (Created in BioRender); (b) reverse transcription‐quantitative polymerase chain reaction analysis of Meis1 mRNA expression in Control and P‐ALI groups; (c) Western blot analysis of Meis1 protein expression in Control and P‐ALI groups; (d) Cell Counting Kit‐8 assay of cell viability in P‐ALI + oe‐NC and P‐ALI + oe‐Meis1 groups; (e) Flow cytometry analysis of apoptosis in P‐ALI + oe‐NC and P‐ALI + oe‐Meis1 groups; (f) Western blot analysis of apoptosis‐related proteins cleaved caspase‐3, Bax, and Bcl2 in both groups; (g) Measurement of intracellular reactive oxygen species (ROS) levels in the two groups; (h) lactate dehydrogenase (LDH) release assay evaluating cytotoxicity in the two groups. All cellular experiments were repeated independently three times. **p < 0.01, ***p < 0.001 and ****p < 0.0001. LPS, lipopolysaccharide; PI, propidium iodide. FITC‐A: fluorescein isothiocyanate area; GAPDH: glyceraldehyde‐3‐phosphate dehydrogenase; OD: optical density.

To further investigate the role of Meis1 in P‐ALI, we generated a stable oe‐Meis1 MLE15 cell line using lentiviral transduction. The overexpression efficiency was experimentally validated, and the modified cells were designated as oe‐Meis1 (Figure S2F,G). Based on the established in vitro P‐ALI model, cells were divided into the P‐ALI + oe‐NC and P‐ALI + oe‐Meis1 groups. CCK‐8 assay showed that Meis1 overexpression significantly improved cell viability, whereas flow cytometry demonstrated a marked reduction in apoptosis compared with the P‐ALI + oe‐NC group (Figure 2d,e). WB analysis further showed that the levels of cleaved caspase‐3 and Bax were significantly decreased, whereas Bcl2 expression was markedly increased in the P‐ALI + oe‐Meis1 group (Figure 2f). These results indicate that Meis1 overexpression effectively attenuated the loss of cell viability and apoptosis induced by phosgene exposure.

Oxidative stress analysis showed that intracellular ROS levels were significantly lower in the P‐ALI + oe‐Meis1 group than in the P‐ALI + oe‐NC group (Figure 2g). In addition, the LDH release assay showed reduced LDH activity in the P‐ALI + oe‐Meis1 group (Figure 2h), indicating alleviated membrane damage. These findings suggest that Meis1 protects alveolar epithelial cells against phosgene‐induced injury, at least in part, by suppressing oxidative stress.

Taken together, the combined analysis of apoptosis‐ and oxidative stress‐related markers indicates that Meis1 exerts a protective effect in P‐ALI by reducing oxidative damage and cell death, thereby preserving alveolar epithelial cell integrity following phosgene exposure.

### 3.3 Successful preparation of Meis1‐Exo@cur for anti‐ALI therapy

Given the critical role of Meis1 in anti‐apoptotic mechanisms, we sought to develop meis1‐engineered mesenchymal stem cell‐derived exosomes (Meis1‐Exos) as a novel biotherapeutic agent to enhance treatment efficacy and minimize P‐ALI. First, we established a stable oe‐Meis1 MSC line by introducing the Meis1 gene via lentiviral vectors (Figure 3a). RT‐qPCR analysis showed that Meis1 mRNA expression was significantly increased in engineered MSCs compared with control MSCs (Figure S3A), and WB analysis further confirmed marked upregulation of Meis1 protein (Figure S3A). These findings confirmed the successful establishment of oe‐Meis1 MSCs.

**FIGURE 3:**
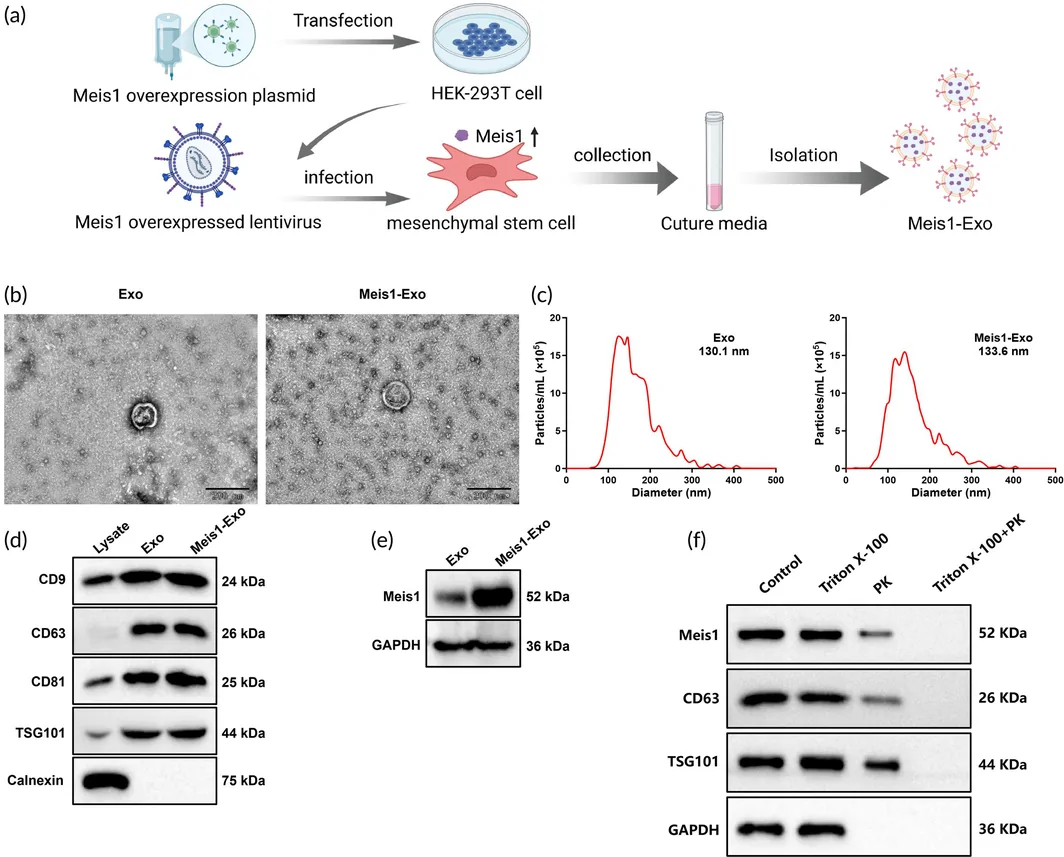
Preparation and characterization of myeloid ecotropic viral integration site 1 (Meis1)‐engineered mesenchymal stem cell (MSCs) and Meis1‐exosomes (Exos). (a) Schematic of lentiviral transduction and Exo extraction from oe‐Meis1 MSCs (Created in BioRender); (b) Transmission electron microscopy (TEM) images showing the morphology of Exos and Meis1‐Exos (scale bar = 200 nm); (c) nanoparticle tracking analysis (NTA) showing the size distribution of Exos and Meis1‐Exos; (d) Western blot analysis of exosomal markers CD9, CD63, CD81, and TSG101 in Exos and Meis1‐Exos; (e) Western blot analysis of Meis1 protein expression in Meis1‐Exos; (f) Protease K protection assay analyzing the localization of Meis1 within exosomes. All experiments were repeated three times. GAPDH: glyceraldehyde‐3‐phosphate dehydrogenase; PK: protease K.

Meis1‐Exos were then isolated from the culture supernatant of modified MSCs by ultracentrifugation and ultrafiltration (Figure 3a). Transmission electron microscopy (TEM) showed that both Exos and Meis1‐Exos exhibited the typical round vesicular morphology with similar diameters (Figure 3b). Nanoparticle tracking analysis (NTA) further demonstrated that both vesicle populations were mainly distributed between 30 and 150 nm, with no significant difference in size distribution (Figure 3c). WB analysis showed that the exosomal markers CD9, CD63, CD81, and TSG101 were expressed in both Exos and Meis1‐Exos, while the negative marker Calnexin was not detected (Figure 3d). The Meis1 protein was also highly expressed in Meis1‐Exos (Figure 3e). To further elucidate the sub‐localization of Meis1 within the engineered Exos, we performed a protease K protection assay (Figure 3f). Meis1 was substantially degraded after treatment with protease K alone, similar to the membrane protein CD63, whereas the luminal protein TSG101 remained protected. Upon membrane disruption with Triton X‐100, all tested proteins were fully degraded. These findings suggest that exogenous Meis1 is predominantly localized on or tightly associated with the exosomal membrane.

Given the reported role of Meis1 in tissue repair and regeneration, and the potent anti‐inflammatory activity of Cur,^31^ we further constructed a Meis1‐Exo‐based drug delivery platform to combine the tissue‐repairing properties of Meis1 with the anti‐inflammatory effects of Cur.

Using an optimized incubation method, Cur was successfully loaded into Meis1‐Exos to generate Meis1‐Exo@Cur (Figure 4a). TEM showed that Meis1‐Exo@Cur retained the typical spherical vesicular morphology (Figure 4b), and NTA demonstrated a size distribution comparable to that of Meis1‐Exos, with particle diameters mainly ranging from 30 to 150 nm (Figure 4c). WB analysis demonstrated that the exosomal markers CD9, CD63, CD81, and TSG101 (Figure S3C) and Meis1 (Figure S3D) were preserved in Meis1‐Exo@Cur. To evaluate cellular uptake, MLE‐15 cells were incubated with 50 μg/mL PKH67‐labeled Meis1‐Exo or Meis1‐Exo@Cur for 24 h. Immunofluorescence staining showed that both Meis1‐Exo and Meis1‐Exo@Cur were efficiently internalized by MLE‐15 cells, and Cur delivered by Meis1‐Exo@Cur was also effectively taken up (Figure 4d,e). Ultraviolet–visible (UV–Vis) spectrophotometry further showed that the drug loading content (DLC) and drug loading efficiency (DLE) of Cur in Meis1‐Exo@Cur were (8.5 ± 0.7)% and (75.2 ± 4.3)%, respectively (Figure 4f).

**FIGURE 4:**
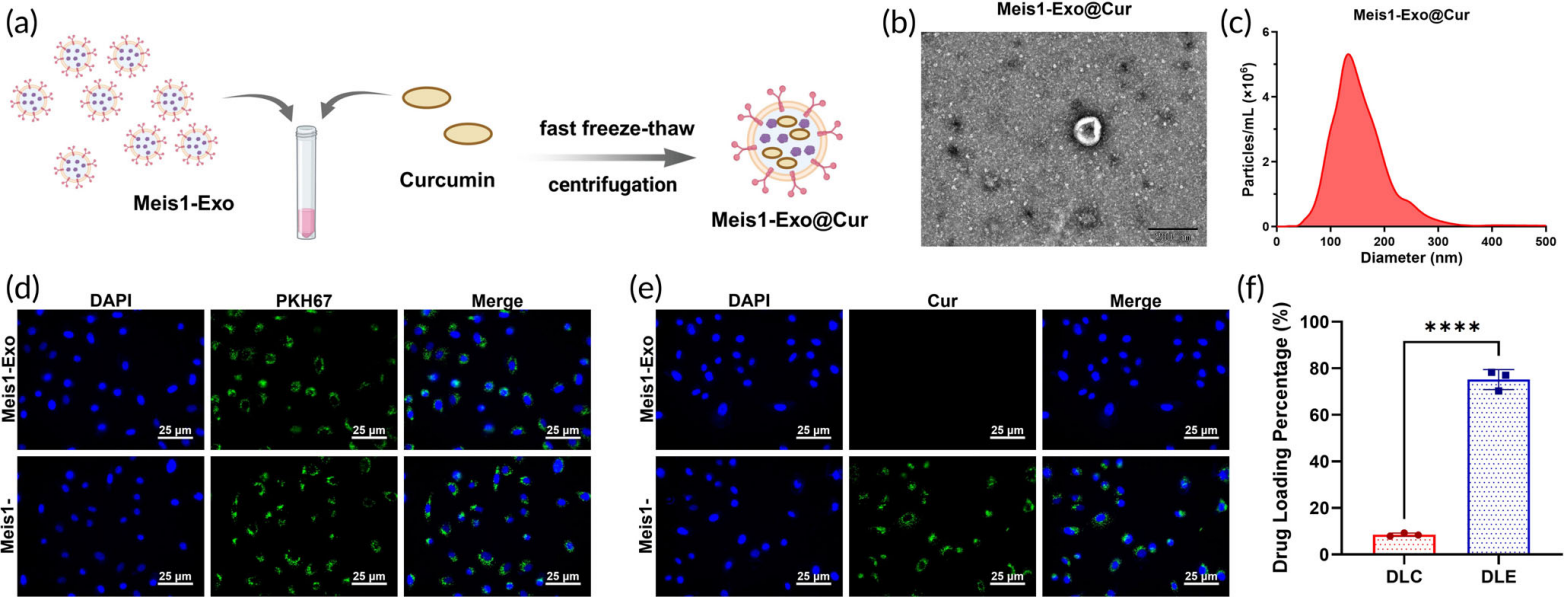
Preparation and characterization of myeloid ecotropic viral integration site 1‐engineered exosomes loaded with curcumin (Meis1‐Exo@Cur). (a) Schematic of the preparation process of Meis1‐Exo@Cur (Created in BioRender); (b) Transmission electron microscopy images showing the morphology of Meis1‐Exo@Cur (scale bar = 200 nm); (c) nanoparticle tracking analysis results showing the particle size distribution of Meis1‐Exo@Cur; (d) immunofluorescence images showing uptake of PKH67‐labeled Meis1‐Exo and Meis1‐Exo@Cur by MLE15 cells (scale bar = 25 μm); (e) immunofluorescence images showing intracellular uptake of curcumin by MLE15 cells (scale bar = 25 μm); (f) bar graph showing the quantitative analysis of the drug loading capacity of Meis1‐Exo@Cur. All experiments were performed in triplicate. ****p < 0.001. Cur, curcumin; DLC, drug loading content; DLE, drug loading efficiency. DAPI: 4',6‐diamidino‐2‐phenylindole.

These results demonstrate the successful construction of Meis1‐Exo@Cur with favorable structural integrity, efficient drug loading, and robust cellular delivery capacity, providing a solid foundation for subsequent therapeutic evaluation.

### 3.4 Meis1‐Exo@cur significantly alleviates phosgene‐induced alveolar epithelial cell injury

We first evaluated the dose‐dependent effects of Meis1‐Exo@Cur using the CCK‐8 assay. Within the concentration range of 12.5–100 μg/mL, Meis1‐Exo@Cur improved the viability of P‐ALI cells in a dose‐dependent manner, with the effect reaching a plateau at 50 μg/mL (Figure 5a). Therefore, 50 μg/mL was selected for subsequent experiments.

**FIGURE 5:**
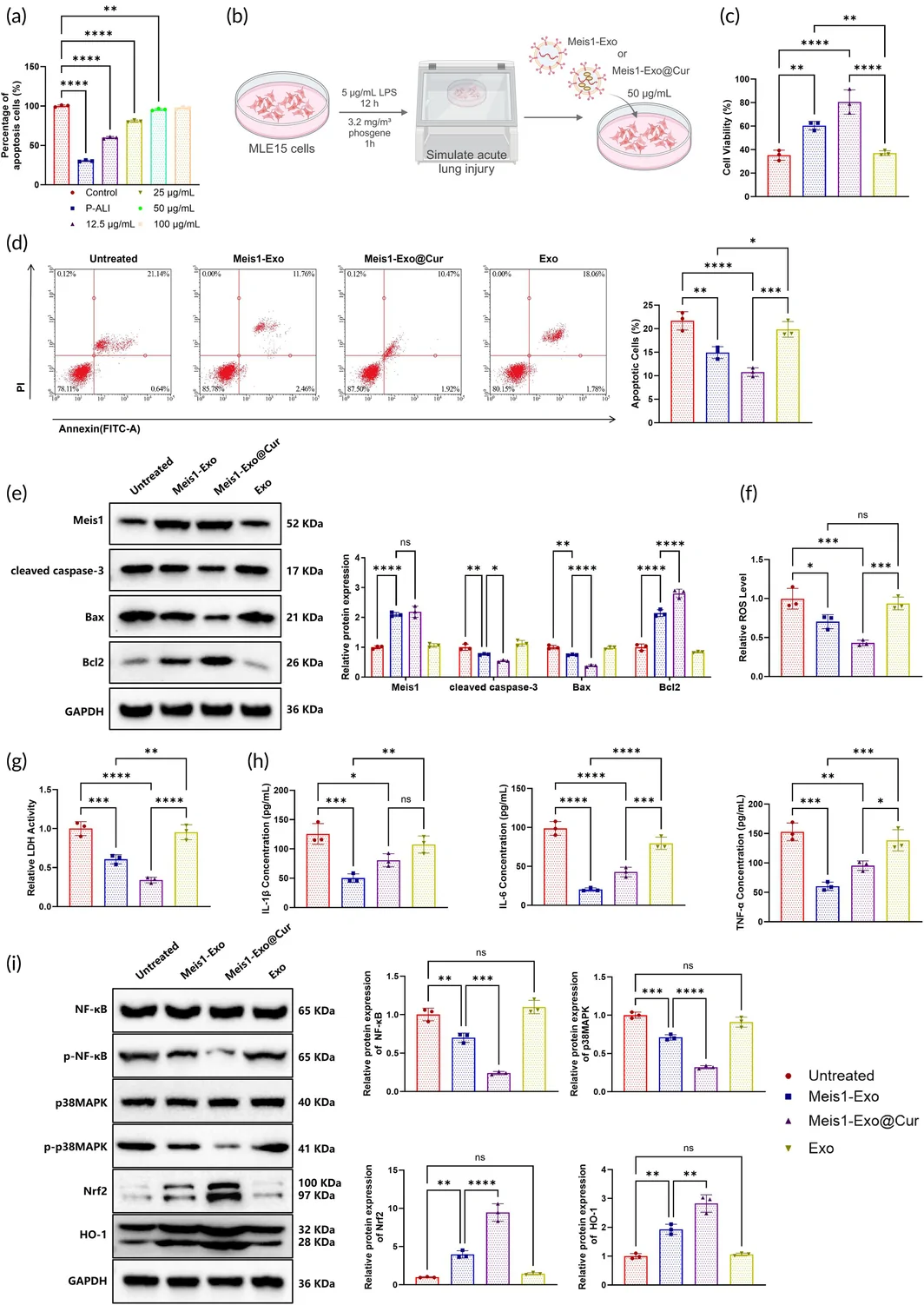
In vitro evaluation of the therapeutic effects of myeloid ecotropic viral integration site 1‐engineered exosomes loaded with curcumin (Meis1‐Exo@Cur) on phosgene‐induced alveolar epithelial cell injury. (a) Dose–response analysis of Meis1‐Exo@Cur in the phosgene‐induced acute lung injury (P‐ALI) model. (b) Schematic workflow of in vitro experiments (Created in BioRender). (c) Evaluation of cell viability in P‐ALI model cells by Cell Counting Kit‐8 assay, presented as relative percentages. (d) Flow cytometry analysis of apoptosis rates in P‐ALI model cells. (e) Western blot analysis of Meis1, cleaved caspase‐3, Bax, and Bcl2 protein expression in P‐ALI model cells. (f) Measurement of intracellular reactive oxygen species levels and lactate dehydrogenase (LDH) activity in P‐ALI model cells to assess oxidative stress and cell damage. (g) LDH release assay to assess cell injury in P‐ALI model cells. (h) Enzyme‐linked immunosorbent assay quantification of inflammatory cytokines TNF‐α, IL‐6, and IL‐1β in cell culture supernatants of P‐ALI model cells. (i) Western Blot analysis of nuclear factor kappa‐B (NF‐κB), mitogen‐activated protein kinase (MAPK), Nrf2, and Heme Oxygenase‐1 (HO‐1) protein expression in P‐ALI model cells. All experiments were performed in triplicate. *ns* indicates no statistical significance (*p* > 0.05); *p < 0.05, **p < 0.01, ***p < 0.001, ****p < 0.0001. Exo, exosome. FITC‐A: fluorescein isothiocyanate area; GAPDH: glyceraldehyde‐3‐phosphate dehydrogenase; HO‐1: heme oxygenase‐1; IL‐6: interleukin‐6; IL‐1β: interleukin‐1 beta; TNF‐α: tumor necrosis factor‐alpha.

After successful construction of Meis1‐Exo@Cur, the in vitro therapeutic effects of different exosome formulations were examined in injured MLE‐15 cells treated with 50 μg/mL Exo, Meis1‐Exo, or Meis1‐Exo@Cur, respectively (Figure 5b). CCK‐8 assay showed that, compared with the Untreated group, the Exo group exhibited only a slight, non‐significant increase in cell viability. In contrast, viability was significantly improved in the Meis1‐Exo group and was further enhanced in the Meis1‐Exo@Cur group (Figure 5c). Flow cytometry analysis showed a similar trend. Although apoptosis in the Exo group was modestly reduced, the difference was not statistically significant. By contrast, the apoptotic rate was significantly decreased in the Meis1‐Exo group and was further reduced in the Meis1‐Exo@Cur group (Figure 5d). Consistently, WB analysis showed that Exo treatment caused only minor, non‐significant changes in apoptosis‐related proteins. In contrast, both Meis1‐Exo and Meis1‐Exo@Cur increased Meis1 protein expression, reduced the levels of cleaved caspase‐3 and Bax, and increased Bcl2 expression, with these effects being more pronounced in the Meis1‐Exo@Cur group (Figure 5e). These findings indicate that both Meis1‐Exo and Meis1‐Exo@Cur exert anti‐apoptotic effects, whereas Cur loading further enhances the therapeutic efficacy.

Oxidative stress analysis further demonstrated that Exo treatment only partially reduced intracellular ROS levels, without statistical significance. In contrast, ROS levels were significantly decreased in the Meis1‐Exo group and showed the greatest reduction in the Meis1‐Exo@Cur group (Figure 5f). Similarly, LDH release assay showed that Exo treatment had only a weak inhibitory effect on LDH activity, whereas LDH release was significantly reduced in the Meis1‐Exo group and further decreased in the Meis1‐Exo@Cur group (Figure 5g). These results suggest that both Meis1‐Exo and Meis1‐Exo@Cur attenuate phosgene‐induced oxidative damage, with Meis1‐Exo@Cur providing superior protection.

Enzyme‐linked immunosorbent assay (ELISA) further showed that Exo treatment slightly reduced the secretion of inflammatory cytokines in the culture supernatant, although the changes were not statistically significant. In contrast, the levels of Tumor Necrosis Factor‐alpha (TNF‐α), Interleukin‐6 (IL‐6), and Interleukin‐1 beta (IL‐1β) were significantly decreased in the Meis1‐Exo group and were further reduced in the Meis1‐Exo@Cur group (Figure 5h). WB results showed that, compared with the Untreated group, the Exo group exerted only minimal effects on suppressing the NF‐κB/MAPK pathway and activating the Nrf2/HO‐1 pathway. Conversely, Meis1‐Exo treatment significantly reduced the phosphorylation of NF‐κB‐ and MAPK‐related proteins while increasing Nrf2 and HO‐1 expression, and these effects were further strengthened by Meis1‐Exo@Cur treatment (Figure 5i).

Collectively, these results further validate that Meis1‐Exo@Cur alleviates P‐ALI by regulating signaling pathways associated with inflammation and oxidative stress.

### 3.5 Meis1‐Exo@cur significantly alleviates P‐ALI in vivo

To further evaluate the in vivo therapeutic efficacy of Meis1‐Exo@Cur, we treated P‐ALI mice via tail vein injection of Exos (Figure 6a). Pulmonary function testing in conscious mice showed that, compared with the Untreated group, the Exo group exhibited only slight, non‐significant improvements in tidal volume, minute ventilation, and respiratory rate. In contrast, tidal volume and minute ventilation were significantly upregulated, whereas respiratory rate was significantly decreased, in the Meis1‐Exo group. These improvements were further enhanced in the Meis1‐Exo@Cur group (Figure 6b). Lung mechanics were subsequently assessed using the FlexiVent system. Compared with the Untreated group, the Exo group exerted only limited effects on the elevated dynamic resistance and reduced lung compliance. However, dynamic resistance was significantly downregulated, and lung compliance was significantly upregulated in the Meis1‐Exo group, with even greater improvement observed in the Meis1‐Exo@Cur group (Figure 6c). *P*–*V* curve analysis further showed that Exo treatment did not significantly reverse the increased RV or decreased TLC induced by phosgene exposure. Conversely, RV was significantly downregulated and TLC significantly upregulated in the Meis1‐Exo group, and these changes were further improved in the Meis1‐Exo@Cur group (Figure 6d). These results indicate that both Meis1‐Exo and Meis1‐Exo@Cur ameliorated phosgene‐induced pulmonary dysfunction, with Meis1‐Exo@Cur showing superior efficacy. H&E staining showed that inflammatory cell infiltration in lung tissues was only mildly reduced in the Exo group, without statistical significance. In contrast, inflammatory infiltration was significantly alleviated in the Meis1‐Exo group and was further reduced in the Meis1‐Exo@Cur group (Figure 6e). Consistently, W/D weight ratio analysis showed that pulmonary edema was markedly increased in the Untreated group. Exo treatment produced only a slight, non‐significant reduction in edema, whereas both Meis1‐Exo and Meis1‐Exo@Cur significantly decreased the W/D ratio (Figure 6f).

**FIGURE 6:**
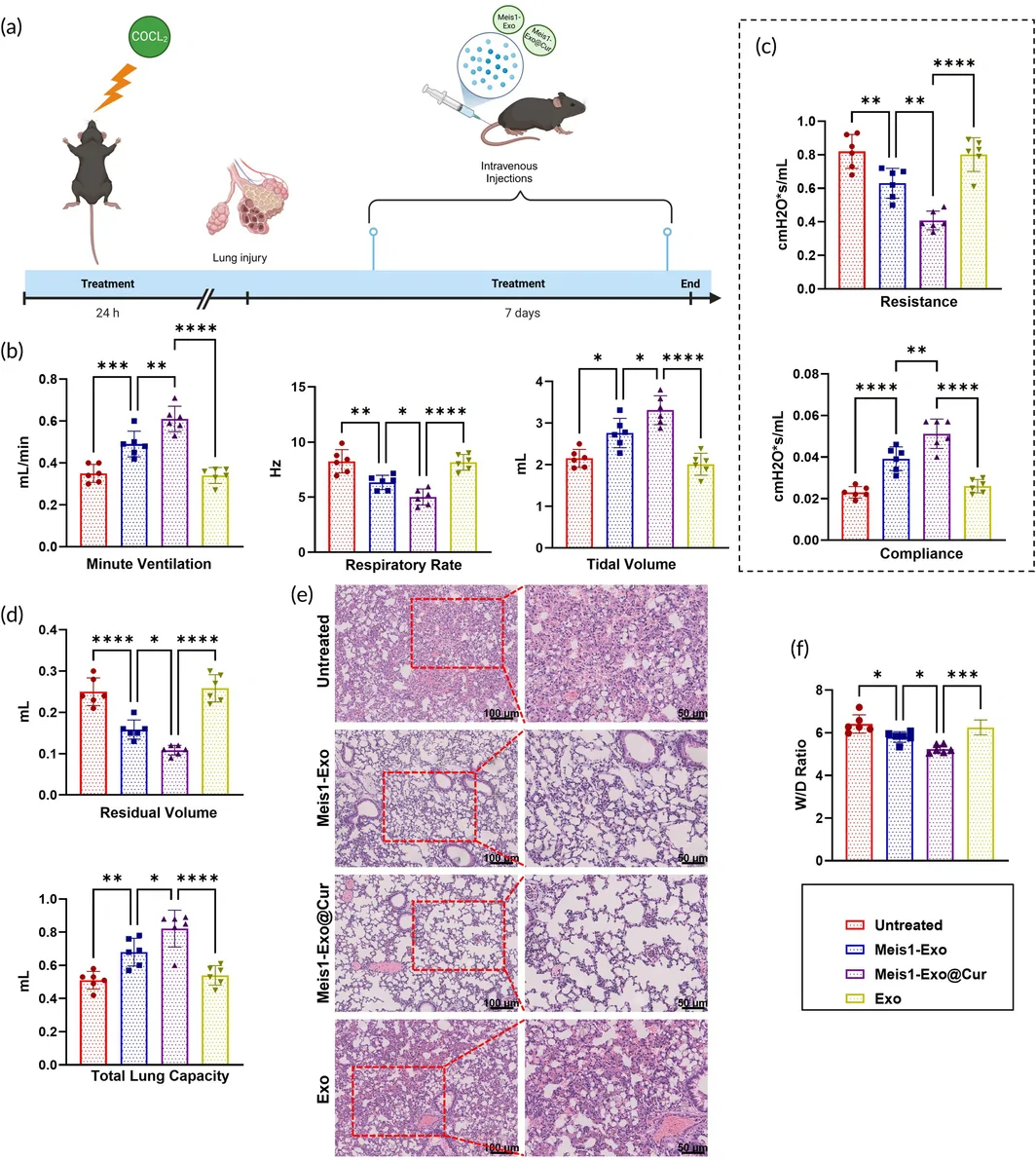
Effects of myeloid ecotropic viral integration site 1‐engineered exosomes loaded with curcumin (Meis1‐Exo@Cur) on pulmonary function in phosgene‐induced acute lung injury (P‐ALI) mice. (a) Schematic of P‐ALI mouse model induction and exosome (Exo)‐based treatment (Created in BioRender); (b) Measurement of tidal volume, respiratory rate, and minute ventilation using a small animal pulmonary function system; (c) Evaluation of dynamic resistance and lung compliance using the FlexiVent system; (d) Calculation of residual volume and total lung capacity; (e) hematoxylin and eosin staining to assess pulmonary inflammatory infiltration; (f) wet‐to‐dry (W/D) to determine pulmonary edema. All experiments were repeated three times with six animals per group. *p < 0.05, **p < 0.01, ***p < 0.001, ****p < 0.0001.

Next, we assessed inflammatory infiltration and cell apoptosis in the lung tissues of each group. ELISA results showed that compared to the Untreated group, the Exo group partially reduced the levels of TNF‐α, IL‐6, and IL‐1β in bronchoalveolar lavage fluid (BALF) and lung tissues, although these changes were not statistically significant. In contrast, these cytokines were significantly decreased in the Meis1‐Exo group and showed the greatest reduction in the Meis1‐Exo@Cur group (Figure 7a). Assessment of oxidative stress further showed that Exo treatment only slightly decreased ROS levels in lung tissues, without statistical significance, whereas ROS levels were significantly reduced in the Meis1‐Exo group and further decreased in the Meis1‐Exo@Cur group (Figure 7b). These findings suggest that although unmodified Exos exerted a modest anti‐inflammatory and antioxidant tendency, Meis1‐Exo and especially Meis1‐Exo@Cur were markedly more effective in suppressing inflammation and oxidative stress in vivo.

**FIGURE 7:**
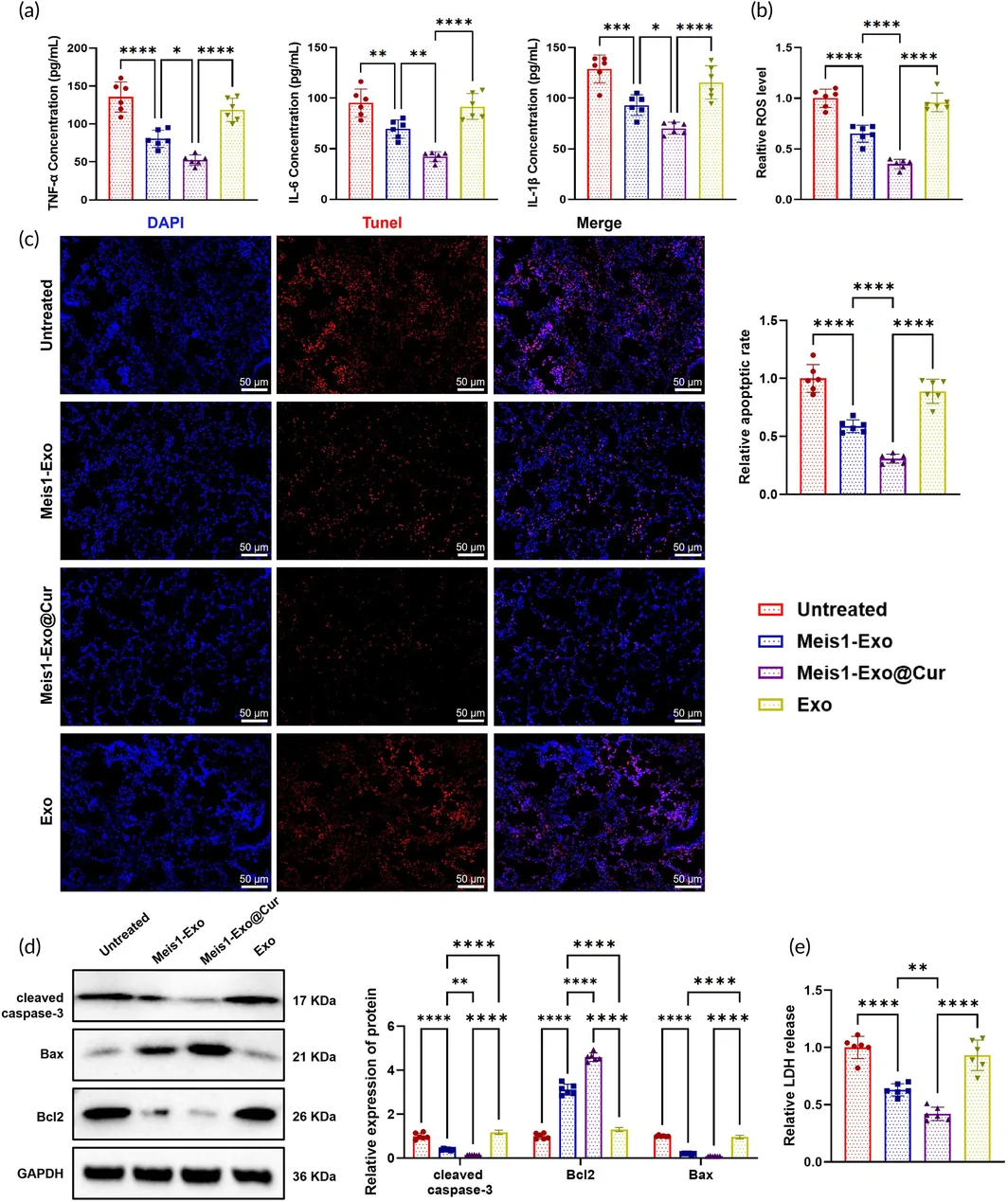
Regulation of inflammation, oxidative stress, and apoptosis by myeloid ecotropic viral integration site 1‐engineered exosomes loaded with curcumin (Meis1‐Exo@Cur) in phosgene‐induced acute lung injury mice. (a) Enzyme‐linked immunosorbent assay analysis of inflammatory cytokines TNF‐α, IL‐6, and IL‐1β in bronchoalveolar lavage fluid and lung tissues; (b) reactive oxygen species (ROS) detection to evaluate oxidative stress levels in lung tissues; (c) terminal deoxynucleotidyl transferase dUTP nick end labeling staining to assess the apoptosis rate in lung tissues; (d) Western Blot analysis of cleaved caspase‐3, Bax, and Bcl2 protein expression in lung tissues; (e) lactate dehydrogenase (LDH) release assay to measure LDH activity in lung tissues. All experiments were repeated three times with six mice per group. *p < 0.05, **p < 0.01, ***p < 0.001, **** *p* < 0.0001. DAPI: 4',6‐diamidino‐2‐phenylindole; GAPDH: glyceraldehyde‐3‐phosphate dehydrogenase; IL‐6: interleukin‐6; IL‐1β: interleukin‐1 beta; TNF‐α: tumor necrosis factor‐alpha.

TUNEL staining showed that lung cell apoptosis displayed only a decreasing trend in the Exo group, without significant difference. In contrast, apoptosis was significantly reduced in the Meis1‐Exo group and was further decreased in the Meis1‐Exo@Cur group (Figure 7c). Consistently, WB analysis demonstrated that Exo treatment induced only weak changes in apoptosis‐related proteins, whereas Meis1‐Exo treatment significantly decreased the expression of cleaved caspase‐3 and Bax while increasing Bcl2 expression, and these effects were even more pronounced in the Meis1‐Exo@Cur group (Figure 7d). The LDH assay showed that, compared with the Untreated group, Exo treatment did not significantly reduce LDH activity, whereas Meis1‐Exo significantly decreased LDH levels, with a further reduction observed in the Meis1‐Exo@Cur group (Figure 7e).

These results indicate that Meis1‐Exo and Meis1‐Exo@Cur exert potent anti‐apoptotic effects in P‐ALI, with Meis1‐Exo@Cur demonstrating the strongest protective outcome.

### 3.6 Meis1‐Exo@cur exhibits favorable in vivo biosafety in the treatment of P‐ALI

To evaluate the in vivo biosafety of Meis1‐Exo@Cur, mice received tail vein injections of Exos and were monitored for body weight changes throughout the treatment period (Figure S4A). No significant differences in body weight were observed among the four groups during the observation period (Figure S4B). Histological examination of major organs (heart, liver, spleen, lungs, and kidneys) revealed no significant pathological alterations in the Meis1‐Exo or Meis1‐Exo@Cur groups compared with the Untreated group (Figure S4C), indicating that neither treatment caused apparent systemic toxicity or organ damage. In addition, serum biochemical analysis showed that the levels of creatine kinase (CK), LDH, alanine aminotransferase (ALT), aspartate aminotransferase (AST), creatinine (CREA), and urea remained within the normal range in both the Meis1‐Exo and Meis1‐Exo@Cur groups relative to the Control group (Figure S5).

These observations reveal that both Meis1‐Exo and Meis1‐Exo@Cur display favorable in vivo biocompatibility and biosafety throughout the course of P‐ALI treatment.

### 3.7 Meis1‐Exo@cur exhibits lung‐targeting distribution characteristics in P‐ALI mice

To evaluate the in vivo distribution of the mesenchymal stem cell‐derived exosome‐based nanodelivery system (Meis1‐Exo@Cur), we intravenously injected the system into P‐ALI model mice via the tail vein and tracked its fluorescent signal at various time points using in vivo and ex vivo organ imaging. After administration of DiR‐labeled Meis1‐Exo@Cur, strong fluorescence signals were detected in the lung region at 1 and 4 h post‐injection (Figure 8a). Quantitative analysis of ex vivo organ imaging further showed that, at 4 h post‐injection, the mean fluorescence intensity (MFI) in lung tissue was significantly higher than that in the heart, liver, spleen, and kidneys (Figure 8b,c), indicating efficient accumulation of the exosome carrier in injured lung tissue.

**FIGURE 8:**
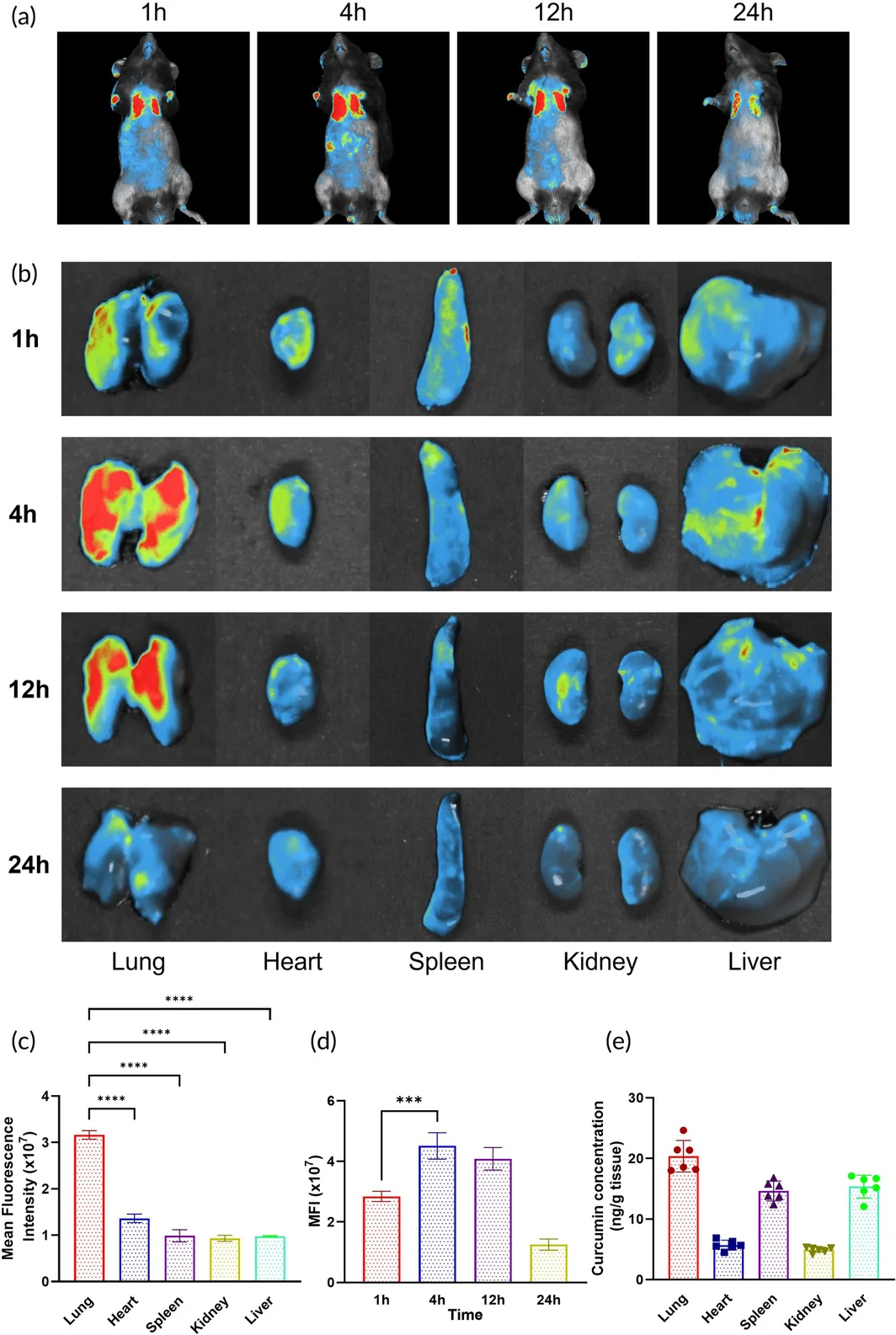
In vivo biodistribution study confirms the lung‐targeted delivery characteristics of myeloid ecotropic viral integration site 1‐engineered exosomes loaded with curcumin (Meis1‐Exo@Cur). (a) Whole‐body fluorescence imaging at different time points (1, 4, 12, and 24 h) following tail vein injection of DiR‐labeled Meis1‐Exo@Cur; (b) Ex vivo fluorescence imaging of major organs (lung, heart, spleen, kidney, and liver); (c) Quantitative analysis of fluorescence intensity in ex vivo organs (lung, heart, spleen, kidney, and liver); (d) Time‐dependent curve of fluorescence intensity in lung tissue; (e) high‐performance liquid chromatography quantitative analysis of curcumin concentration in various organs at 4 h post‐injection. Data are presented as mean ± standard deviation (SD) with three animals per group (*n* = 3). ***p < 0.001, **** *p* < 0.0001. MFI, mean fluorescence intensity.

We next assessed the pulmonary retention kinetics of the nanosystem. As shown in Figure 8d, the fluorescence signal in the lungs reached its peak at 4 h after injection and remained at a relatively high level for up to 12 h, followed by a gradual decline. This prolonged retention suggests an extended therapeutic window in the injured lung.

To determine whether Cur displayed a distribution pattern consistent with that of the exosome carrier, Cur concentrations in major organs were further quantified by high‐performance liquid chromatography (HPLC). At 4 h post‐injection, Cur levels in lung tissue were significantly higher than those in all other examined organs (Figure 8e). This result was consistent with the fluorescence imaging data, demonstrating that Meis1‐Exo@Cur effectively and selectively delivered Cur to the lungs, which may contribute to its enhanced therapeutic efficacy in vivo.

### 3.8 Meis1‐Exo@cur alleviate P‐ALI by regulating inflammatory cytokines and immune infiltration

To elucidate the molecular mechanisms underlying the therapeutic effects of Meis1‐Exo@Cur, we performed RNA‐seq on lung tissues collected from both the Meis1‐Exo@Cur treatment group and the P‐ALI model group (Figure 9a). Differential expression analysis identified 2275 significant DEGs, including 1159 downregulated and 1116 upregulated genes, indicating substantial transcriptional reprogramming after treatment. Notably, several key pro‐inflammatory cytokines, including IL‐1α, IL‐1β, IL‐6, and TNF‐α, were significantly downregulated in the Meis1‐Exo@Cur group, while Meis1 expression was markedly upregulated (Figures 9b and S6A). Correlation analysis further showed that Meis1 expression was strongly negatively correlated with IL‐6 and TNF‐α levels (Figure 9c), suggesting that the therapeutic effect of Meis1‐Exo@Cur is partly associated with suppression of inflammatory cytokine expression.

**FIGURE 9:**
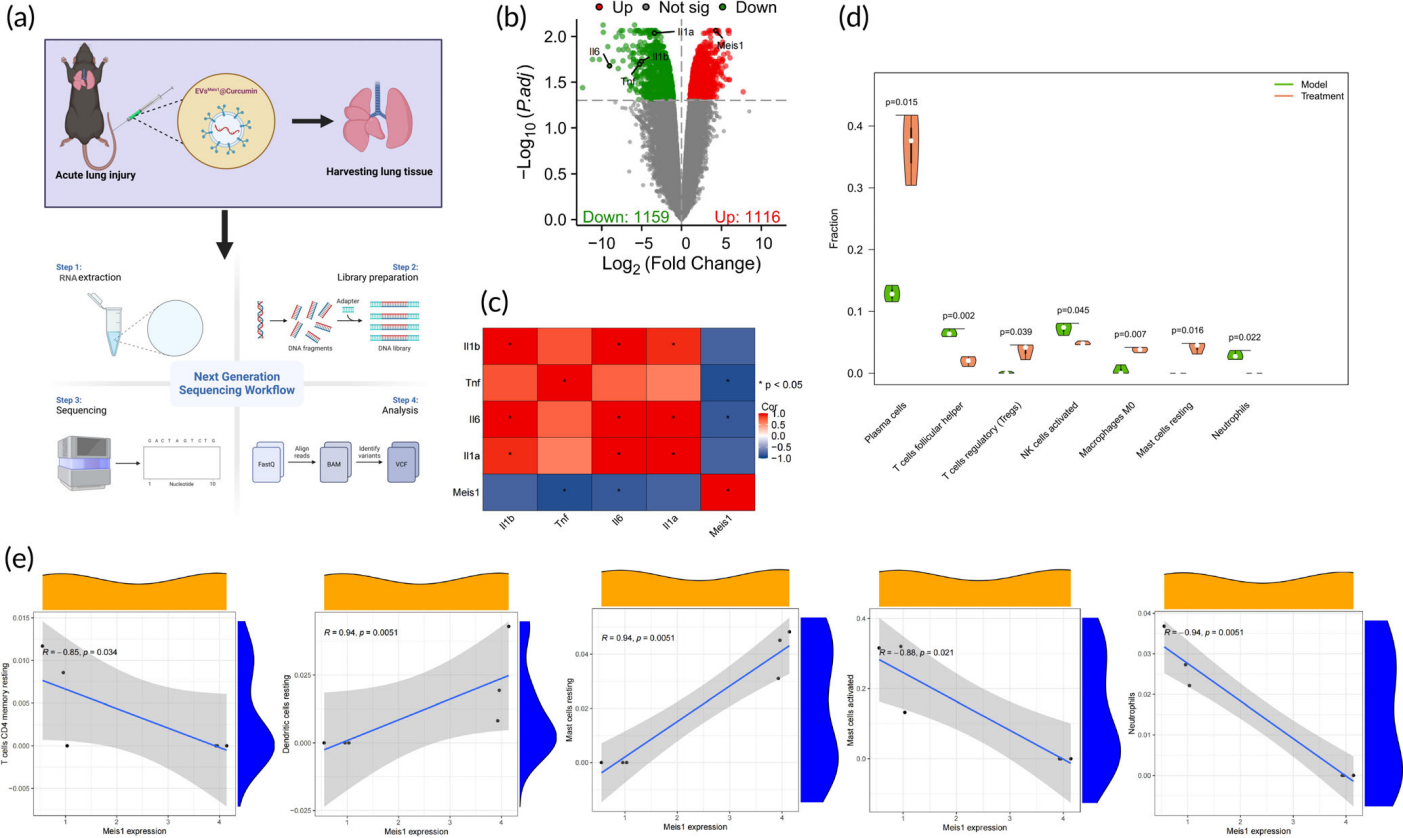
Mechanistic exploration of immune microenvironment regulation by myeloid ecotropic viral integration site 1‐engineered exosomes loaded with curcumin (Meis1‐Exo@Cur) in phosgene‐induced acute lung injury (P‐ALI).(a) Workflow of RNA sequencing analysis (Created in BioRender); (b) Volcano plot of differential gene expression, with red indicating significantly upregulated genes, green indicating downregulated genes, and gray representing non‐significant genes; (c) Heatmap showing the correlation between Meis1 and inflammatory cytokines; (d) Violin plot of immune cell composition differences between the model and treatment lung tissue samples; (e) Correlation scatter plots between Meis1 expression and immune cell subpopulations. Model: *N* = 3; Treatment: *N* = 3.

To further characterize treatment‐induced changes in the immune microenvironment, the CIBERSORT algorithm was used to analyze immune cell infiltration in the two groups. Compared with the P‐ALI group, the Meis1‐Exo@Cur group showed significantly increased proportions of plasma cells, regulatory T cells (Tregs), M0 macrophages, and resting mast cells, whereas the proportions of follicular helper T cells, activated natural killer (NK) cells, and neutrophils were significantly decreased (Figures 9d and S6B,C).

Single‐gene correlation analysis further demonstrated that Meis1 expression was significantly negatively correlated with CD4 memory resting T cells, activated mast cells, and neutrophils, but positively correlated with resting dendritic cells and resting mast cells (Figure 9e). These findings suggest that Meis1‐Exo@Cur may promote lung repair and inflammation resolution, at least in part, by reshaping immune cell infiltration and activation patterns.

In summary, these results indicate that Meis1‐Exo@Cur exerts therapeutic effects in P‐ALI by suppressing pro‐inflammatory cytokine expression and remodeling the immune infiltration landscape.

### 3.9 In vivo immunophenotyping confirms Meis1‐Exo@cur reshapes the lung injury immune microenvironment

To directly validate the immune alterations suggested by the transcriptomic and CIBERSORT analyses, we performed in vivo flow cytometric analysis of key immune cell populations in lung tissues (Figure 10a). Using specific antibody markers, we quantitatively analyzed the proportions of neutrophils, macrophage subtypes, and Tregs within the lung immune cell population (Figure 10b).

**FIGURE 10:**
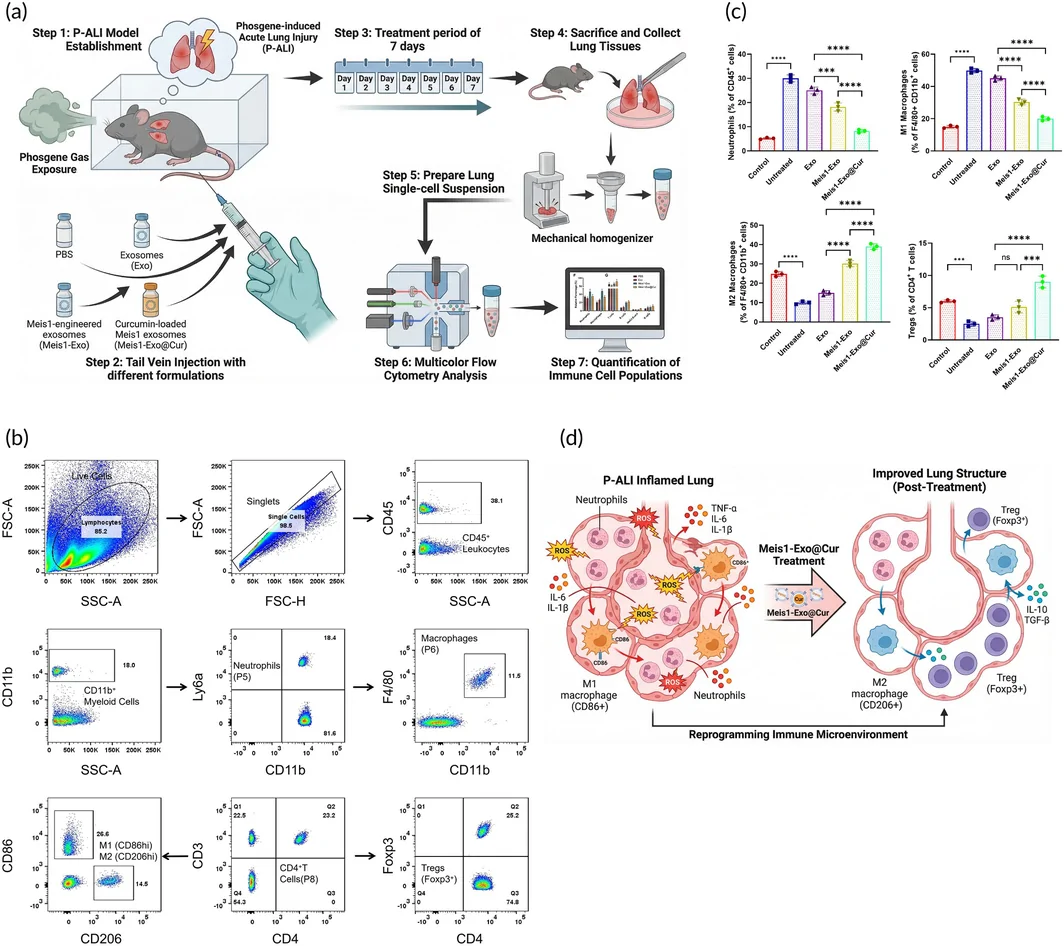
In vivo flow cytometric validation of immune microenvironment remodeling by myeloid ecotropic viral integration site 1‐engineered exosomes loaded with curcumin (Meis1‐Exo@Cur) in phosgene‐induced acute lung injury (P‐ALI). (a) Schematic workflow of the in vivo immunophenotyping validation experiment; (b) Gating strategy for flow cytometric analysis of immune cells in lung tissues; (c) Quantitative analysis of the proportions of key immune cell populations in lung tissues across different experimental groups; (d) Schematic diagram illustrating the mechanism by which Meis1‐Exo@Cur remodels the lung immune microenvironment. Data are presented as mean ± standard deviation (SD), with individual scatter points representing independent mouse samples (*n* = 6 per group). Statistical significance was determined using one‐way analysis of variance followed by Tukey's post hoc test. ***p < 0.001, **** *p* < 0.0001. FSC‐H: forward scatter height; SSC‐A: side scatter area.

Compared with the Control group, the proportions of neutrophils and pro‐inflammatory M1 macrophages were significantly increased in the Untreated group (Figure 10c). Following Meis1‐Exo@Cur treatment, the infiltration of both cell populations was markedly reduced to near‐baseline levels, with greater efficacy than that observed in the Exo and Meis1‐Exo groups. In contrast, the proportions of tissue‐repair‐associated M2 macrophages and immunosuppressive Tregs were decreased in the Untreated group but significantly increased after Meis1‐Exo@Cur treatment (Figure 10c).

These in vivo immunophenotyping data were highly consistent with the CIBERSORT predictions and further supported the reduced inflammatory infiltration observed by H&E staining. Together, these findings indicate that the therapeutic effects of Meis1‐Exo@Cur extend beyond suppression of inflammatory cytokine expression and involve active remodeling of the immune microenvironment in injured lungs. Specifically, Meis1‐Exo@Cur attenuated the excessive innate inflammatory response characterized by neutrophils and M1 macrophages, while promoting tissue repair and immunoregulatory programs associated with M2 macrophages and Tregs (Figure 10d). This immunophenotypic shift provides an important cellular basis for Meis1‐Exo@Cur‐mediated lung repair.

### 3.10 Meis1‐Exo@cur alleviate ALI by regulating MAPK and NF‐κB signaling pathways

To further elucidate the mechanisms underlying the therapeutic effects of Meis1‐Exo@Cur, we performed comparative proteomic analysis of lung tissues from the model group and the Meis1‐Exo@Cur‐treated group (Figure S7A). A total of 3231 proteins were identified, among which 54 were significantly differentially expressed (logFC >1, *p* < 0.05), including 29 upregulated and 25 downregulated proteins. The overall distribution of DEPs was visualized by volcano plot (Figure S7B), and their expression patterns across groups were further illustrated by heatmap analysis (Figure S7C).

To investigate the relationship between transcriptomic and proteomic alterations, we performed an overlap analysis between DEGs and DEPs, which identified 27 overlapping molecules (Figure S7D). These candidates are likely to participate in the key signaling events associated with the therapeutic effects of Meis1‐Exo@Cur. KEGG enrichment analysis of the overlapping genes revealed significant enrichment in the MAPK, NF‐κB, and TNF signaling pathways, as well as necroptosis and apoptosis (Figure S7E), suggesting that Meis1‐Exo@Cur may exert its protective effects by modulating inflammatory and cell death‐related signaling cascades.

GO enrichment analysis further showed that the DEPs were significantly associated with the I‐kappaB kinase/NF‐κB signaling pathway, membrane microdomains, growth factor receptor binding, and NF‐κB binding (Figure S7F). These findings further support the KEGG results and provide additional functional evidence for the molecular mechanisms underlying treatment efficacy.

Taken together, these results suggest that Meis1‐Exo@Cur alleviates ALI by regulating key pathways involved in inflammation and cell survival, particularly the MAPK and NF‐κB signaling pathways, while also suppressing necroptosis and apoptosis.

## 4 DISCUSSION

This study demonstrates that Meis1 is significantly downregulated in P‐ALI and to uncover its critical regulatory role in alveolar epithelial cell injury. Previous studies have mainly focused on the functions of Meis1 in the cardiovascular system, hematopoietic stem cell maintenance, and metabolic regulation, whereas its role in pulmonary diseases, particularly ALI, has remained largely unclear. In the present study, Meis1 was identified as a potential key gene in P‐ALI through transcriptomic analysis and PPI network screening, and its function was subsequently validated in vitro. Meis1 overexpression markedly reduced apoptosis and ROS accumulation in alveolar epithelial cells exposed to phosgene and lipopolysaccharide (LPS), indicating a protective role in oxidative stress‐related injury. These findings expand the known functional repertoire of Meis1 and, for the first time, establish its relevance in acute pulmonary inflammatory injury, providing a new perspective on the molecular mechanisms underlying P‐ALI.

In recent years, MSC‐Exos have attracted increasing attention as important mediators of intercellular communication. Owing to their low immunogenicity, favorable biocompatibility, and intrinsic targeting potential, MSC‐Exos have shown promising therapeutic value in various inflammatory diseases.^15^, ^32^, ^33^ In ALI, MSC‐Exos have been reported to suppress pro‐inflammatory cytokine release and restore immune homeostasis, thereby alleviating lung injury.^11^, ^20^ However, the therapeutic efficacy of native Exos may be limited by variability in their bioactive cargo, which can compromise their stability and targeting efficiency.^10^ In this study, we addressed this limitation by engineering MSCs via lentiviral transduction to overexpress Meis1, thereby generating Meis1‐Exos that also served as carriers for Cur. This dual‐functional design significantly improved therapeutic efficacy. The combination of engineered Exos with drug delivery represents an emerging strategy in exosome‐based therapy and reflects a transition from broad regenerative effects toward more precise molecular intervention. Notably, the unmodified Exo group showed substantially weaker protective effects than the Meis1‐Exo group, directly indicating that Meis1 overexpression is a key determinant of the enhanced anti‐inflammatory and anti‐apoptotic properties of the engineered Exos, rather than a simple extension of their basal reparative function.

Furthermore, the efficient and sustained pulmonary accumulation of Meis1‐Exo@Cur observed in the P‐ALI model provides direct in vivo evidence for the targeting capability of the mesenchymal stem cell‐derived exosome‐based delivery platform. This pharmacokinetic profile is particularly important because it ensures that both therapeutic cargoes, Meis1 and Cur, achieve and maintain effective local concentrations at the site of injury, thereby facilitating their synergistic anti‐inflammatory, antioxidant, and anti‐apoptotic effects. These findings support the rationale for selecting MSC‐derived Exos as delivery vehicles by taking advantage of their intrinsic tropism toward injured tissues, as suggested in previous studies.^15^ More importantly, the successful lung‐targeted delivery observed here demonstrates the feasibility of using engineered MSC‐derived Exos for the co‐delivery of proteins and hydrophobic small‐molecule drugs, providing a promising strategy to overcome major pharmacological limitations such as poor bioavailability and insufficient tissue specificity.

Cur, a natural polyphenolic compound, has demonstrated anti‐inflammatory, antioxidant, and anti‐apoptotic effects in various lung disease models.^34^, ^35^, ^36^ However, its clinical application has been greatly limited by poor bioavailability, rapid metabolism, and insufficient tissue targeting.^37^, ^38^, ^39^ In the present study, Cur was delivered to injured lung tissue using Meis1‐engineered Exos, which markedly improved its stability and bioavailability. Compared with previous delivery strategies, the Meis1‐Exo@Cur system developed here showed clear advantages in delivery efficiency, cellular uptake, and tissue targeting, thereby substantially enhancing the biological activity of Cur in P‐ALI. These findings provide a novel strategy for the efficient utilization and precise delivery of natural therapeutic compounds. In addition, this study is the first to demonstrate the lung‐targeting distribution characteristics of Meis1‐Exo@Cur in a P‐ALI mouse model through in vivo imaging and quantitative drug analysis. Following intravenous administration, both the exosomal carrier and Cur showed the highest accumulation in lung tissue, with retention spanning the critical therapeutic window. This distribution pattern provides a direct pharmacokinetic basis for the pronounced therapeutic efficacy observed, as efficient pulmonary delivery ensures effective local drug concentrations, thereby enabling more potent regulation of the NF‐κB/Nrf2 pathways, suppression of inflammation, and promotion of tissue repair.

Multi‐omics integration has emerged as a powerful strategy for elucidating disease mechanisms.^27^ This study combined transcriptomic and proteomic data to identify 27 key regulatory genes at the intersection of DEGs and DEPs. Further, GO and KEGG pathway enrichment analyses revealed the involvement of classical inflammatory and antioxidant signaling pathways, including MAPK, NF‐κB, and Nrf2. CIBERSORT‐based immune infiltration analysis showed that several immune cell subsets, such as neutrophils, monocytes, and M1 macrophages, were significantly reduced in the treatment group, indicating that Meis1‐Exo@Cur can modulate the immune microenvironment. To directly validate these bioinformatic predictions, key immune cell populations in lung tissues were further quantified by in vivo flow cytometry. The results confirmed that Meis1‐Exo@Cur significantly reshaped the immune microenvironment in P‐ALI. Specifically, the reduction in neutrophils and M1 macrophages was closely associated with decreased levels of pro‐inflammatory cytokines (e.g., TNF‐α and IL‐6), whereas the increased proportions of M2 macrophages and Tregs were linked to enhanced anti‐inflammatory signaling and activation of tissue‐repair programs.^40^, ^41^ This shift in immune phenotype from “pro‐inflammatory” to “reparative” constitutes the cytological basis for the dual anti‐inflammatory and pro‐regenerative efficacy of Meis1‐Exo@Cur. In addition, annotation of DEPs revealed that the treatment group exhibited upregulation of proteins related to membrane microdomain stability and growth factor receptor binding, offering important insights into the mechanisms underlying alveolar barrier protection. By integrating multi‐omics approaches, this study systematically explored the therapeutic mechanisms of Meis1‐Exo@Cur at the levels of genes, proteins, signaling pathways, and immune cell dynamics, thereby enhancing the credibility, completeness, and mechanistic clarity of the findings.

Flow cytometric analysis revealed a nuanced picture regarding cell death dynamics. While Meis1‐Exo@Cur treatment significantly reduced the overall apoptosis rate, the proportion of early apoptotic cells (Annexin V^+^ PI^−^) within the population increased relative to the injury model group, whereas the proportion of late apoptotic/necrotic cells (Annexin V^+^ PI^+^) decreased drastically. Rather than being contradictory, this pattern suggests that Meis1‐Exo@Cur profoundly influences the progression and mode of cell death. Under severe phosgene‐induced injury, cells may rapidly lose membrane integrity in response to intense oxidative stress and inflammatory insult, thereby progressing directly into late apoptosis or necrosis. In the present study, the synergistic antioxidant and anti‐inflammatory actions of Meis1‐Exo@Cur, including activation of the Nrf2/HO‐1 pathway and inhibition of NF‐κB signaling, may have attenuated the initial injury burden and preserved membrane integrity during the early phase of cell death. As a result, a subset of cells that would otherwise undergo rapid disintegration may instead have shifted toward a more orderly apoptotic process. Such a transition from necrotic or terminally damaged states to early‐stage apoptosis is considered beneficial for tissue repair because apoptotic cells can be cleared efficiently by macrophages through “find‐me” and “eat‐me” signals, thereby avoiding the exaggerated secondary inflammation caused by the release of necrotic cellular contents.^42^, ^43^ Moreover, the significant reduction in cleaved caspase‐3 expression and the overall decrease in apoptosis observed in this study confirm the strong anti‐apoptotic effect of Meis1‐Exo@Cur at the execution stage of cell death, whereas the relative enrichment of early apoptotic cells may reflect its regulatory effect on upstream death progression. This finding is consistent with emerging views that effective tissue‐repair strategies should not only reduce the extent of cell death but also redirect residual cell death toward forms that are more compatible with resolution of inflammation and tissue recovery.

The NF‐κB and MAPK signaling pathways are central regulators of the inflammatory response and have become key therapeutic targets in ALI. In contrast, the Nrf2/HO‐1 pathway plays a vital role in antioxidant defense and cytoprotection.^44^, ^45^, ^46^ Although previous studies have shown that modulation of any one of these pathways can partially alleviate inflammation, single‐target interventions often produce limited therapeutic benefit.^47^, ^48^ In this study, we demonstrated that Meis1‐Exo@Cur not only inhibited NF‐κB pathway activation and suppressed the expression of pro‐inflammatory cytokines such as IL‐1β and TNF‐α but also upregulated Nrf2 and its downstream target HO‐1, thereby reducing ROS production. These molecular changes were accompanied by reduced apoptosis in vitro, marked histopathological improvement in lung tissues, and significant recovery of pulmonary function in vivo. These findings indicate that Meis1‐Exo@Cur exerts dual regulatory effects by simultaneously attenuating inflammation and promoting tissue repair. This integrated pathway modulation may represent a promising therapeutic strategy for ALI.

Conventional therapies for ALI, including glucocorticoids, antibiotics, and antioxidants, generally act through limited molecular targets and are frequently associated with suboptimal efficacy and considerable side effects.^49^, ^50^ In contrast, Meis1‐Exo@Cur integrates the advantages of targeted delivery and multi‐pathway regulation. Its therapeutic efficacy is reflected not only in the suppression of inflammation and oxidative stress, but also in the activation of endogenous repair programs that improve alveolar structure and function. In addition, engineered Exos can be customized according to specific pathological conditions, which increases their flexibility across different disease settings. As an emerging therapeutic platform, engineered Exos may have broad application prospects in acute respiratory distress syndrome, pulmonary fibrosis, and even systemic inflammatory disorders, and represent a promising frontier in extracellular vesicle‐based gene‐drug combination therapy.

Despite the encouraging therapeutic efficacy of Meis1‐Exo@Cur demonstrated through multidimensional analyses, several limitations of this study should be acknowledged. First, all in vivo experiments were conducted exclusively in male C57BL/6 mice. Although this design helped minimize variability associated with hormonal fluctuations and improved consistency in the initial mechanistic investigation, it did not address potential sex‐specific differences in immune responses, drug metabolism, and tissue repair, which may limit the generalizability of the findings across sexes. Second, the present work was primarily based on a P‐ALI model, and whether the therapeutic effects of Meis1‐Exo@Cur can be extended to other forms of lung injury, such as inhalation injury or infectious pneumonia, remains to be determined. Third, although significant changes in immune cell proportions were identified, the precise functional contributions of individual immune cell subsets, including specific T cell populations and dendritic cells, as well as the upstream signaling mechanisms driving these immunological changes, require further investigation, potentially using cell‐specific genetic models. Fourth, the robust effects observed in cellular and animal experiments still require validation in larger‐scale preclinical studies and, ultimately, in clinical settings. Finally, the delivery strategy and long‐term safety of Meis1‐Exo@Cur need to be further optimized and systematically evaluated before clinical translation can be considered. Future studies should therefore focus on improving exosome stability, enhancing targeting efficiency, conducting comprehensive biosafety assessments, and performing rigorous preclinical validation to facilitate the translation of this therapeutic platform from experimental research to clinical application in ALI and related pulmonary diseases.

## 5 CONCLUSION

This study systematically evaluated the therapeutic efficacy and molecular mechanisms of Meis1‐Exo@Cur in P‐ALI. Transcriptomic analysis revealed that Meis1 was significantly downregulated in P‐ALI, and its overexpression effectively attenuated lung injury by reducing oxidative stress and apoptosis. Meis1‐Exo@Cur exerted enhanced therapeutic effects by inhibiting the NF‐κB signaling pathway and activating the Nrf2/HO‐1 pathway, thereby decreasing the levels of inflammatory cytokines (TNF‐α, IL‐6, and IL‐1β), enhancing antioxidant capacity, and promoting lung tissue repair. In vitro experiments showed that Meis1‐Exo@Cur significantly improved cell viability, reduced apoptosis, and alleviated oxidative stress. In vivo studies further confirmed that Meis1‐Exo@Cur improved pulmonary function, reduced pulmonary edema, and exhibited favorable biosafety. These findings highlight the therapeutic potential of Meis1‐Exo@Cur in P‐ALI through coordinated regulation of inflammation, oxidative stress, and apoptosis‐related pathways.

In addition, this study successfully developed a novel nanotherapeutic platform by loading Cur into Meis1‐engineered Exos. This system exhibited marked anti‐inflammatory, antioxidant, and tissue‐repair‐promoting effects in the P‐ALI model. Its therapeutic efficacy was associated with coordinated modulation of multiple signaling pathways, including NF‐κB, MAPK, and Nrf2/HO‐1, leading to reduced inflammatory cytokine production, inhibition of apoptosis, attenuation of oxidative stress, and remodeling of the immune microenvironment. These molecular and cellular effects translated into substantial improvement in lung function and tissue injury. Collectively, this study proposes a novel therapeutic strategy for P‐ALI and provides a potential framework for exosome‐based drug delivery systems in the precise treatment of other inflammatory lung diseases.

## AUTHOR CONTRIBUTIONS

**Ke Pang:** Conceptualization; investigation; methodology; validation; writing – original draft. **Zhao Yang:** Conceptualization; supervision; project administration; writing – review and editing; funding acquisition. **Zefa Ling:** Formal analysis; investigation; visualization. **Shouyong Wang:** Resources; investigation. **Bo Hu:** Data curation; formal analysis; validation. **Yukun Zhang:** Methodology; investigation; data curation; writing – review and editing. **Surong Deng:** Supervision; validation; writing – review and editing.

## FUNDING INFORMATION

This study was supported by the Natural Science foundation of Chongqing Yongchuan Project (2025yc‐cxfz10053) and the Chongqing Science‐Health Joint Medical Research Project (2025ZDXM003).

## CONFLICT OF INTEREST STATEMENT

The authors declare no conflict of interest.

## Supporting information


**Data S1.** Supporting Information.

**Data S2.** Supporting Information.

## Data Availability

All data generated or analyzed during this study are included in this article and/or its Supporting Information S1 and S2 files. Further enquiries can be directed to the corresponding author.
