# Supplementary material for: Innovative myeloid ecotropic viral integration site 1‐engineered exosome platform combating oxidative stress and inflammation in acute pulmonary damage
Source: Bioeng Transl Med. 2026 May 14;11(5):e70150. doi: 10.1002/btm2.70150 (PMC13636224; doi:10.1002/btm2.70150)
Supplement: Supplementary file 1 [file BTM2-11-e70150-s001.docx]

**Materials and Methods**

**Bulk RNA-seq**

Total RNA was extracted from mouse lung tissues using TRIzol reagent (Invitrogen, USA). Samples were collected from the Control group (n = 3), P-ALI group (n = 3), and Meis1-Exo@Cur treatment group (n = 3). RNA concentration and purity were measured using a NanoDrop 2000 spectrophotometer (Thermo Fisher Scientific, USA), and RNA integrity was assessed using an Agilent 2100 Bioanalyzer (Agilent Technologies, USA), with RNA integrity number (RIN) values > 7.0 considered acceptable. Complementary DNA (cDNA) libraries were prepared using the TruSeq RNA Sample Preparation Kit (Illumina, USA). Briefly, RNA was reverse-transcribed into cDNA, followed by end repair, A-tailing, adaptor ligation, and PCR amplification. Library quality was further evaluated using the Agilent 2100 Bioanalyzer. Qualified libraries were subjected to paired-end sequencing on the Illumina HiSeq 2500 platform, generating approximately 50 million 150-bp paired-end reads per sample.

Raw sequencing reads were first subjected to quality assessment using FastQC (v0.11.9). Low-quality reads and adaptor sequences were removed using Trimmomatic (v0.39). Clean reads were then aligned to the reference genome using HISAT2 (v2.2.1), and gene expression levels were quantified using HTSeq (v0.11.2).

**Differential Expression Analysis**

Differentially expressed genes (DEGs) were identified from the RNA-seq dataset using the Limma package in R, with thresholds set at |log_2_FoldChange| > 1 and *p* < 0.05. The DEGs were visualized using volcano plots generated with the ggplot2 package. All data analyses were conducted using R version 4.3.1 (R Foundation for Statistical Computing).

**Gene Ontology (GO) and Kyoto Encyclopedia of Genes and Genomes (KEGG) Enrichment Analysis**

GO enrichment analysis was performed in R using the org.Hs.eg.db annotation package (version 3.1.0) together with the clusterProfiler package (version 3.14.3). Genes were mapped to the reference annotation database, and functional enrichment was conducted based on the background gene set. KEGG pathway enrichment analysis was performed using clusterProfiler, with the latest pathway annotations retrieved through the KEGG REST API. The gene set size was restricted to a minimum of 5 genes and a maximum of 5000 genes. Statistical significance was defined as *p* < 0.05 and false discovery rate (FDR) < 0.25. Enrichment results were visualized using ggplot2 in R.

**Least Absolute Shrinkage and Selection Operator (LASSO) Regression**

LASSO regression analysis was performed using the glmnet package in R. Ten-fold cross-validation was applied to identify key genes associated with disease status. The glmnet function was used with L1 regularization (α = 1), and 100 lambda values were tested. The optimal lambda value was selected according to the minimum cross-validated deviance (lambda.min). Genes with non-zero coefficients at the optimal lambda were considered key candidate genes.

**Random Forest-Based Feature Selection**

A random forest model was constructed using the randomForest package in R to evaluate gene importance based on RNA-seq data from all six samples. Samples were classified into the Control and P-ALI groups. The number of trees (ntree) was set to 500. Model performance was assessed using an error-rate plot as the number of trees increased, and the optimal tree number was determined using the which.min() function based on the minimum error rate. Gene importance was evaluated using Mean Decrease Gini and Mean Decrease Accuracy, with higher values indicating greater importance.

**Correlation Heatmap Analysis**

Pairwise correlation analysis among variables was conducted using Pearson’s correlation coefficient. The results were visualized as a heatmap using the ggplot2 package in R. Statistical significance was denoted as *p* < 0.05. Correlation strength was interpreted according to the absolute value of the correlation coefficient, with values closer to 1 indicating stronger associations.

**Immune Infiltration Analysis**

Immune cell infiltration was estimated from gene expression profiles using the CIBERSORT algorithm in combination with the e1071 and preprocessCore packages in R. A total of 22 immune cell subtypes were quantified. All statistical analyses and data visualizations were performed in R version 4.2.1, and figures were generated using the ggplot2 package.

**Proteomic Analysis**

Proteomic profiling was performed using a Q Exactive mass spectrometer (Thermo Fisher Scientific, USA). Total protein was extracted and quantified with a BCA Protein Assay Kit (Thermo Fisher Scientific, USA). Protein samples were separated by sodium dodecyl sulfate-polyacrylamide gel electrophoresis (SDS-PAGE), followed by in-gel digestion with trypsin (Promega, USA). The resulting peptides were separated using an EASY-nLC 1000 liquid chromatography system (Thermo Fisher Scientific, USA) and then analyzed by mass spectrometry. Raw data were processed using MaxQuant software, and protein identification was conducted against the UniProt database with a FDR threshold of <1%. Label-free quantification (LFQ) was used for protein quantification. Proteins with |log_2_ fold change| > 1 and *p* < 0.05 (Student’s t-test) were considered significantly differentially expressed.

**Venn Diagram**

To visually represent the overlap between DEGs and DEPs, a Venn diagram was generated using the VennDiagram package (version 1.6.20) in R.

**Establishment and Grouping of the Cell Model**

The mouse type II alveolar epithelial cell line MLE-15 (Mus musculus, male, lung alveolar epithelium; RRID: CVCL_0459; catalog no. CSC-I9200L, USA) was used to investigate the effects of phosgene exposure on alveolar epithelial cells. Cells were cultured in RPMI-1640 medium (Gibco, USA) supplemented with 10% fetal bovine serum (FBS; Gibco, USA) and 1% penicillin-streptomycin (Sigma-Aldrich, USA) until reaching approximately 80% confluence. To establish the acute lung injury (ALI) model *in vitro*, cells were first treated with 5 μg/mL lipopolysaccharide (LPS; Sigma-Aldrich, USA) for 12 h, followed by exposure to 3.2 mg/m^3^ phosgene for 1 h. Phosgene gas was generated by adding N,N-dimethylformamide (Macklin, Shanghai, China) to a hexachloroethane solution. To evaluate the therapeutic effects of Meis1-Exo and Meis1-Exo@Cur and to clarify the specific role of Meis1, injured cells were divided into four groups: Untreated group (model only), Exo group (treated with 50 μg/mL unmodified mesenchymal stem cell-derived exosomes after injury), Meis1-Exo group (treated with 50 μg/mL Meis1-engineered exosomes after injury), and Meis1-Exo@Cur group (treated with 50 μg/mL curcumin-loaded Meis1-engineered exosomes after injury) (PMID: 37673367).

**Cell Counting Kit-8 (CCK-8) Assay**

Cell viability was assessed using the CCK-8 (Dojindo, Japan) according to the manufacturer’s instructions. After 48 h of treatment, 10 μL of CCK-8 solution was added to each well. Following incubation at 37 °C for 30 min, absorbance was measured at 450 nm using a microplate reader (Bio-Rad, USA).

**Flow Cytometry Analysis**

Cell apoptosis was evaluated using the Annexin V-FITC/PI Apoptosis Detection Kit (C1062L, Beyotime, China). Cells were seeded in 6-well plates at a density of 1 × 10^6^ cells per well. After treatment, cells were collected and resuspended in 195 μL Annexin V-FITC binding buffer, followed by the addition of 5 μL Annexin V-FITC and 10 μL PI. Samples were incubated in the dark at room temperature for 15 min and analyzed within 20 min using a BD FACSCalibur flow cytometer. The apoptosis rate was calculated as the percentage of Annexin V-positive cells.

**RT-qPCR for Gene Expression**

Total RNA was extracted from tissues and cells using TRIzol reagent (Thermo Fisher Scientific, USA). cDNA was synthesized using the PrimeScript RT Reagent Kit (Takara, Japan). Reverse transcription-quantitative polymerase chain reaction (RT-qPCR) was performed using the SYBR Premix Ex Taq II Kit (Takara, Japan) on an ABI 7500 Real-Time PCR System (Thermo Fisher Scientific, USA). The amplification protocol consisted of an initial denaturation at 95 °C for 30 s, followed by 40 cycles of 95 °C for 5 s and 60 °C for 30 s. Melting curve analysis was then performed at 95 °C for 15 s, 60 °C for 60 s, and 95 °C for 15 s. GAPDH was used as the internal reference gene. Each sample was analyzed in triplicate, and all experiments were independently repeated three times. Relative gene expression levels were calculated using the 2^-ΔΔCt^ method. Primer sequences are listed in Table S1.

**DCF-DA Staining**

Intracellular reactive oxygen species (ROS) levels were measured using 2′,7′-dichlorofluorescein diacetate (DCF-DA) staining. Cells were seeded in black 96-well plates and treated as indicated. After treatment, 10 μM DCF-DA (Sigma-Aldrich, USA) was added to each well, followed by incubation at 37 °C in 5% CO₂ for 30 min. During incubation, DCF-DA was deacetylated by intracellular esterases and subsequently oxidized by ROS to generate the fluorescent product DCF. Fluorescence intensity was measured at excitation/emission wavelengths of 485/528 nm using a fluorescence microplate reader (BioTek, USA) to quantify intracellular ROS levels.

**Western Blot Analysis**

Total protein was extracted from cells or tissues using RIPA lysis buffer (89901, Thermo Fisher, USA). Protein concentrations were determined using a BCA Protein Assay Kit (23225, Thermo Fisher, USA). Equal amounts of protein (30 µg per sample) were separated by SDS-PAGE and transferred to PVDF membranes (IPFL00010, Millipore, USA). Membranes were blocked with 5% non-fat milk (LP0033B, Thermo Fisher, USA) and incubated overnight at 4 °C with primary antibodies against Meis1, cleaved-caspase-3, Bcl2, Bax, CD9, CD63, CD81, TSG101, Calnexin, and GAPDH (antibody details are listed in Table S2). After washing, membranes were incubated with HRP-conjugated anti-rabbit IgG secondary antibody (7074, Cell Signaling Technology, USA). Protein bands were visualized using an ECL substrate (WP20005, Thermo Fisher, USA) and detected with a chemiluminescence imaging system (Bio-Rad, USA). Band intensities were quantified using ImageJ software (version 1.52, NIH, USA).

**Lactate Dehydrogenase (LDH) Release Assay**

Cell membrane integrity and cytotoxicity were assessed using a LDH assay kit (Roche, Switzerland) according to the manufacturer’s instructions. After treatment, culture supernatants were collected and transferred to 96-well plates. LDH reaction mixture was added to each well and incubated in the dark at room temperature for 30 min. Absorbance was then measured at 490 nm using a microplate reader (Bio-Rad, USA). LDH activity in each group was expressed as a percentage relative to the Control group. Each condition was tested in triplicate.

**Lentiviral Transduction Assay**

To investigate the potential protective role of Meis1 in phosgene-induced alveolar epithelial injury, lentiviral transduction was used to achieve Meis1 overexpression. The control plasmid oe-NC and the target plasmid oe-Meis1 (constructed using the pCDH vector), along with the packaging plasmids pMD2.G (Addgene #12259) and psPAX2 (Addgene #12260), were co-transfected into HEK-293T cells using the Lentiviral Packaging Kit (V48820, Invitrogen). After 48 h, the viral supernatant was collected and concentrated using a Lentivirus Concentration Kit (631231, Takara, Japan). MLE15 cells were infected with the lentivirus, and transduced cells were selected using 10 μg/mL puromycin (540222, Sigma-Aldrich) for at least one week to establish stable cell lines. Cells were divided into two groups: one transduced with oe-Meis1 (P-ALI + oe-Meis1 group) and the other with the empty vector (P-ALI + oe-NC group). Both groups were pretreated with 5 μg/mL LPS (L5293, Sigma-Aldrich, USA) for 12 h and then exposed to 3.2 mg/m^3^ phosgene for 1 h to simulate ALI. All plasmid constructs were provided by HANBIO (Shanghai, China).

**Preparation and Characterization of Meis1-Exos**

To generate Meis1-engineered exosomes (Meis1-Exos), the Meis1 overexpression construct was introduced into mouse umbilical cord-derived mesenchymal stem cells (MSCs; Pricella, China) using a lentiviral vector (pCDH, System Biosciences, USA). Cells were cultured in complete medium (Pricella, China), and Polybrene (Sigma-Aldrich, USA) was added to enhance transduction efficiency. Twenty-four hours after transduction, cells were selected in medium containing 2 μg/mL puromycin (Sigma-Aldrich, USA) to establish a stable Meis1-overexpressing MSC line. Overexpression of Meis1 was confirmed by RT-qPCR and western blot analysis.

Exosomes were isolated from culture supernatants collected after 48 h of incubation with Meis1-engineered MSCs. Briefly, the supernatants were subjected to ultracentrifugation at 100,000 × g for 2 h at 4 °C (Beckman Coulter, USA), followed by purification using 100-kDa ultrafiltration membranes (Millipore, USA). All procedures were performed at 4 °C to maintain sample stability. As a control, primary MSCs without Meis1 lentiviral transduction were cultured under identical conditions, and exosomes were isolated using the same procedure. These unmodified exosomes were designated as Exo and used for comparative experiments.

**Preparation and Characterization of Meis1-Exo@Cur**

Curcumin (Cur), dissolved in ethanol, was mixed with Meis1-Exos at a fixed mass ratio of 1:4 and incubated at room temperature for 15 min. The mixture then underwent 2–3 rapid freeze–thaw cycles to facilitate drug loading. Unbound Cur was removed by centrifugation at 5,000 × g for two cycles. The resulting curcumin-loaded exosome complex, designated Meis1-Exo@Cur, was collected either by centrifugation at 14,000 × g for 3 h or using a commercial exosome isolation reagent (Total Exosome Isolation Reagent, Thermo Fisher Scientific, USA).

The concentration of free Cur in the supernatant was measured using a UV–Vis spectrophotometer (Shimadzu, Japan) at 425 nm. Drug loading content (DLC) and drug loading efficiency (DLE) were calculated as follows: DLC (%) = amount of loaded drug / (amount of loaded drug + amount of nanoparticles used) × 100%; DLE (%) = amount of loaded drug / total amount of drug added × 100%. Encapsulation efficiency and loading capacity were determined according to the initial amounts of exosomes and Cur used.

The morphology and particle size of Meis1-Exo@Cur were examined by transmission electron microscopy (TEM; FEI, USA). For TEM analysis, a small volume of exosome suspension was placed onto copper grids, negatively stained with 2% phosphotungstic acid (pH 7.0) for 5 min, and then observed under the microscope. Nanoparticle tracking analysis (NTA) was performed using a NanoSight NS300 system (Malvern Panalytical, UK) to determine particle size distribution and to assess the effect of Cur loading on exosome properties.

**Protease K Protection Assay**

To determine the spatial localization of Meis1 within engineered exosomes, a protease K protection assay was performed. A total of 20 μg Meis1-Exo was divided into four equal portions and subjected to the following treatments: Control group, with an equal volume of phosphate-buffered saline (PBS); Triton X-100 group, treated with 0.1% (v/v) Triton X-100; Protease K group, treated with 100 μg/mL protease K; and Triton X-100 + Protease K group, treated with both 0.1% Triton X-100 and 100 μg/mL protease K. All samples were incubated at 37 °C for 30 min. Phenylmethanesulfonyl fluoride (PMSF) was then added to a final concentration of 2 mM to terminate protease activity. After treatment, the protein levels of Meis1, the luminal exosomal marker tumor susceptibility gene 101 (TSG101), and the membrane marker CD63 were analyzed by western blot.

**Cellular Uptake Assay**

To evaluate the uptake efficiency of Meis1-Exo@Cur by MLE15 cells (CRL-2110, ATCC, USA), exosomes were labeled with the fluorescent dye PKH67 (PKH67GL, Sigma-Aldrich, USA). Labeled exosomes were co-cultured with cells at a final concentration of 50 μg/mL for 24 h. Subsequently, cells were fixed with 4% paraformaldehyde and stained with DAPI (10 μg/mL, D3571, Thermo Fisher, USA) for 10 min at room temperature. The intracellular localization of exosomes and Curcumin was then observed and analyzed using a laser scanning confocal microscope (Leica Microsystems, Germany).

**Establishment of Phosgene-Induced Acute Lung Injury Mouse Model**
Thirty-six healthy adult male C57BL/6 mice (Vital River Laboratory Animal Technology Co., Ltd., Beijing, China) were used in this study. After stratification by body weight, mice were randomly assigned into five groups (n = 6 per group) using a random number table: Control, Untreated (P-ALI model without treatment), Exo (P-ALI model treated with unmodified mesenchymal stem cell-derived exosomes), Meis1-Exo (P-ALI model treated with Meis1-engineered exosomes), and Meis1-Exo@Cur (P-ALI model treated with curcumin-loaded Meis1-engineered exosomes). Group allocation was performed by investigators not involved in subsequent procedures, and all experimental assessments were conducted in a blinded manner in accordance with the ARRIVE guidelines (Table S3). Mice were housed in a specific pathogen-free (SPF) environment with a controlled temperature of 22°C, 50% relative humidity, and ad libitum access to standard rodent chow and water. Except for the Control group, the other four groups were exposed to phosgene (8.33 mg/m³) for 10 minutes to establish the P-ALI model; the Control group was maintained under identical conditions without phosgene exposure. Treatment commenced 24 hours post-modeling. Mice in the Exo, Meis1-Exo, and Meis1-Exo@Cur groups received daily tail vein injections of the respective exosome formulations (50 μg/mouse, dissolved in 50 μL PBS) for 7 consecutive days. The Control and Untreated groups received equivalent volumes of PBS injections during the same period. All animal procedures adhered to blinding principles, were performed by researchers not involved in grouping, and complied with ARRIVE reporting guidelines.

General health status was monitored by tracking changes in body weight. At the end of the experiment, major organs, including the heart, liver, spleen, lungs, and kidneys, were harvested and subjected to hematoxylin and eosin (H&E) staining for histopathological evaluation. Serum samples were collected, and levels of creatine kinase (CK), LDH, alanine aminotransferase (ALT), aspartate aminotransferase (AST), creatinine (CREA), and urea were measured using a fully automated biochemical analyzer (Hitachi 7180, Japan) to assess hepatic and renal function.

**Flow Cytometric Analysis of Immune Cell Infiltration in Lung Tissue**
To quantitatively analyze immune cell infiltration in lung tissue, three mice from each group were randomly selected and sacrificed under deep anesthesia 24 hours after the final administration. Whole lungs were rapidly excised and placed in ice-cold PBS containing 2% FBS. Lung tissues were thoroughly minced with ophthalmic scissors and digested using a Mouse Lung Dissociation Kit (e.g., Miltenyi Biotec, GentleMACS™ Dissociator, or a similar mechanical/enzymatic digestion method). The digested cell suspension was filtered through a 70 μm cell strainer, and red blood cells were lysed using Red Blood Cell Lysis Buffer (e.g., BioLegend, Cat. #420301). The resulting single-cell suspension was resuspended in PBS containing 2% FBS for cell counting and viability assessment (trypan blue exclusion method, viability >85%).
For each sample, 1×10^6^ cells were used for surface and intracellular marker staining. First, Fc receptors were blocked with anti-mouse CD16/32 antibody (clone 93, BioLegend) for 10 minutes at room temperature. Subsequently, a cocktail of fluorochrome-conjugated antibodies was added and incubated for 30 minutes at 4°C in the dark: APC/Cy7 anti-mouse CD45, FITC anti-mouse CD11b, PE/Cy7 anti-mouse Ly6G, PerCP/Cy5.5 anti-mouse F4/80, BV421 anti-mouse CD86, APC anti-mouse CD206, PE anti-mouse CD3, and BV605 anti-mouse CD4 (Table S4). After surface staining, cells were washed twice with staining buffer. For regulatory T cell (Treg) detection, fixation and permeabilization were performed using the Foxp3/Transcription Factor Staining Buffer Set. Intracellular staining was then carried out by adding BV785 anti-mouse Foxp3 antibody and incubating for 60 minutes at room temperature in the dark. Following staining, cells were resuspended in flow cytometry staining buffer. All samples were acquired on a flow cytometer equipped with 405nm, 488nm, 561nm, and 640nm lasers. Data analysis was performed using FlowJo software (Version 10.8, BD Life Sciences).

***In Vivo* Biodistribution Study**
To evaluate the *in vivo* targeted delivery characteristics of Meis1-Exo@Cur, a time-dependent biodistribution study was conducted using a separate cohort of P-ALI model mice independent of the efficacy evaluation experiments described above. Meis1-Exo@Cur (50 μg), labeled with the near-infrared fluorescent dye DiR (Thermo Fisher Scientific, USA), was injected intravenously via the tail vein into P-ALI mice. A total of 12 mice were used, with three mice allocated to each time point (1, 4, 12, and 24 h; Table S3). Whole-body fluorescence imaging was performed at the indicated time points using an in vivo imaging system (IVIS Spectrum, PerkinElmer, USA). After imaging, mice were euthanized, and the heart, liver, spleen, lungs, and kidneys were collected for ex vivo organ imaging and quantitative analysis of mean fluorescence intensity (MFI). To quantify curcumin distribution, curcumin-loaded Meis1-Exo@Cur was injected in the same manner. Tissue homogenates were collected at the same time points, and curcumin concentration (ng/g tissue) was measured by high-performance liquid chromatography (HPLC, Agilent 1260 Infinity II).

**Pulmonary Function Testing**

Pulmonary function in awake mice was first assessed by barometric whole-body plethysmography to measure respiratory rate, tidal volume, and minute ventilation. Each mouse was placed in a sealed chamber maintained under fully humidified conditions. Pressure fluctuations generated during the respiratory cycle, together with chamber temperature, were recorded and used to calculate respiratory parameters based on the corresponding waveforms.

Mice were then anesthetized, subjected to tracheostomy, and intubated with an 18-gauge cannula. Mechanical ventilation was performed using the FlexiVent system (SCIREQ, Canada) at a respiratory rate of 150 breaths/min, a tidal volume of 0.25 mL, and a positive end-expiratory pressure (PEEP) of 3 cm H₂O. Following a deep inflation to 30 cm H_2_O, total respiratory resistance and compliance were measured within 1 min. The lungs were then degassed and re-expanded, and quasi-static pressure-volume (P-V) curves were obtained using the FlexiVent system. Total lung capacity (TLC) and residual volume (RV) were calculated from the P-V curve data.

**H&E Staining**

To assess histopathological changes and inflammatory cell infiltration in lung tissues after phosgene exposure, H&E staining was performed. Paraffin-embedded lung tissue sections were deparaffinized, rehydrated through a graded alcohol series, and stained with hematoxylin for 2 min and eosin for 1 min. Sections were then dehydrated through 70%, 80%, 90%, 95%, and absolute ethanol, cleared twice in xylene for 5 min each, and mounted with neutral resin. Histopathological changes were observed under a light microscope (Aperio VERSA, Leica Biosystems).

**Lung Wet-to-Dry Weight Ratio (W/D)**

Pulmonary edema was evaluated using the lung W/D ratio. After euthanasia, the lungs were excised and extrapulmonary tissues were carefully removed. A small section of the left lung (2 mm × 2 mm × 2 mm) was immediately weighed to obtain the wet weight. The tissue was then dried at 60 °C for 96 h and reweighed to obtain the dry weight. The W/D ratio was calculated to reflect the degree of pulmonary edema.

**Bronchoalveolar Lavage Fluid (BALF) Collection**

Following euthanasia, BALF was collected through tracheal cannulation. Prewarmed sterile saline (37 °C) was slowly instilled into the lungs in two aliquots and retained for approximately 30 s before withdrawal. The lavage procedure was repeated 2–3 times while carefully avoiding tissue injury and overinflation. A total recovery rate of at least 70% was considered acceptable. The recovered BALF was collected into sterile centrifuge tubes and maintained at 4 °C. Samples were immediately centrifuged at 300 × g for 10 min at 4 °C to separate the supernatant from the cell pellet. The supernatant was used for subsequent inflammatory cytokine analysis.

**TUNEL Staining**

TUNEL staining was conducted to detect apoptotic cells in lung tissues using a commercial TUNEL Apoptosis Detection Kit (C1089, Beyotime, China). Paraffin-embedded tissue sections were deparaffinized in xylene, rehydrated through a graded ethanol series, and treated with proteinase K (20 μg/mL; Beyotime, China) at 20–37 °C for 15–30 min. Subsequently, 50 μL TUNEL reaction mixture was added to each section, followed by incubation at 37 °C for 60 min in the dark. Sections were then counterstained with 4′,6-diamidino-2-phenylindole (DAPI; Thermo Fisher Scientific, USA) for 10 min at room temperature. TUNEL-positive cells were visualized using a fluorescence microscope (Olympus, Japan).

**Enzyme-Linked Immunosorbent Assays (ELISA) Assay**

To quantify inflammatory cytokines, cell culture supernatants were collected after treatment and centrifuged at 3,000 rpm for 10 min at 4 °C to remove debris and suspended cells. The clarified supernatants were transferred to fresh tubes and stored at −80 °C until analysis. For lung tissue assays, tissues were rinsed with ice-cold PBS, snap-frozen in liquid nitrogen, homogenized in lysis buffer containing protease inhibitors, and centrifuged at high speed at 4 °C to obtain the supernatant. Cytokine levels were measured using mouse-specific ELISA kits for TNF-α (D721150-0048), IL-6 (D721022-0048), and IL-1β (D721017-0048) (R&D Systems, USA). Samples and standards were incubated in precoated microplates for 2 h, followed by washing and sequential incubation with biotinylated detection antibodies for 1 h and horseradish peroxidase-conjugated streptavidin for 30 min. Tetramethylbenzidine (TMB) substrate was then added for color development, and the reaction was terminated with stop solution. Absorbance was measured at 450 nm using a microplate reader (BioTek, USA), and cytokine concentrations were calculated from standard curves.

**Statistical Analysis**

All experiments were independently repeated at least three times. Data are presented as mean ± standard deviation (SD). Normality was assessed using the Shapiro–Wilk test. The sample size for animal experiments was determined with reference to commonly used group sizes in comparable murine lung injury studies, preliminary observations, and the ethical principles of animal experimentation (3R principles). Accordingly, six mice per group were used for the primary efficacy experiments. Additional animals were included to account for potential losses during model establishment and experimental procedures. Detailed animal allocation is provided in Table S3.

For normally distributed data, two-group comparisons were performed using Student’s t-test, while comparisons among multiple groups were conducted using one-way analysis of variance (ANOVA) followed by Tukey’s HSD post hoc test. For data that did not meet assumptions of normality or homogeneity of variance, nonparametric tests were applied, including the Mann–Whitney U test or Kruskal–Wallis test. All statistical analyses were performed using GraphPad Prism 9.5.0 (GraphPad Software, Inc.) and R version 4.2.1 (R Foundation for Statistical Computing). A two-sided *p* value < 0.05 was considered statistically significant.
