# Supplementary material for: Innovative myeloid ecotropic viral integration site 1‐engineered exosome platform combating oxidative stress and inflammation in acute pulmonary damage
Source: Bioeng Transl Med. 2026 May 14;11(5):e70150. doi: 10.1002/btm2.70150 (PMC13636224; doi:10.1002/btm2.70150)
Supplement: Supplementary file 2 [file BTM2-11-e70150-s002.docx]

**Figure S1. Validation of the P-ALI mouse model.** (A) Measurement of tidal volume, respiratory rate, and minute ventilation in Control and P-ALI mice using a small animal pulmonary function testing system; (B) Assessment of dynamic resistance and compliance using the FlexiVent system; (C) Calculation of RV and TLC; (D) H&E staining showing inflammatory infiltration and alveolar structural damage in lung tissues (scale bar = 50 µm); (E) W/D to evaluate pulmonary edema; (F) TUNEL staining to assess apoptosis in lung tissues (scale bar = 50 µm). All experiments were repeated three times with three animals per group. **p* < 0.05, ***p* < 0.01, ****p* < 0.001.

**Figure S2. *In vitro* P-ALI model and validation of Meis1 overexpression.** (A) Relative cell viability of Control and P-ALI groups assessed by CCK-8 assay (expressed as a percentage relative to the Control group); (B) Flow cytometry analysis of apoptosis rates in Control and P-ALI groups; (C) Western Blot analysis of apoptosis-related proteins cleaved caspase-3, Bax, and Bcl2; (D) Intracellular ROS levels in Control and P-ALI cells; (E) LDH levels in the culture supernatants of Control and P-ALI cells; (F) RT-qPCR analysis confirming Meis1 mRNA expression in oe-Meis1 cells; (G) Western Blot validation of Meis1 protein overexpression in oe-Meis1 cells. All cellular experiments were performed in triplicate. **p* < 0.05, ***p* < 0.01, ****p* < 0.001.

**Figure S3. Supplemental characterization of Meis1-engineered MSCs and Meis1-Exo@Cur.**(A) RT-qPCR analysis of Meis1 mRNA expression in oe-Meis1 MSCs; (B) Western Blot analysis of Meis1 protein expression in oe-Meis1 MSCs; (C) Western Blot analysis of exosomal markers CD9, CD63, CD81, and TSG101 in Meis1-Exo@Cur; (D) Western Blot validation of Meis1 protein expression in Meis1-Exo@Cur. All experiments were performed in triplicate. **p* < 0.05, ***p* < 0.01, ****p* < 0.001.

**Figure S4. Effects of Meis1-Exo and Meis1-Exo@Cur treatment on mouse body weight and major organs.** (A) Experimental workflow for animal treatment (Created in BioRender); (B) Monitoring of mouse body weight during intravenous administration of Exo treatments; (C) H&E staining of major organs (heart, liver, spleen, lung, kidney) to evaluate histopathological changes. Each group contained six animals. **p* < 0.05, ***p* < 0.01, ****p* < 0.001.

**Figure S5. Effects of Meis1-Exo and Meis1-Exo@Cur on serum biochemical parameters.** (A) Automated biochemical analyzer measurements of CK, LDH, ALT, AST, CREA, and urea serum levels. Each group contained six animals.

**Figure S6. RNA-seq analysis.** (A) Heatmap showing the top 30 DEGs; (B) Composition of 22 immune cell subtypes in lung tissues from model and treatment groups; (C) Violin plot illustrating the differences in immune cell infiltration between lung tissue samples from the Model and Treatment groups. Model: n = 3; Treatment: n = 3.

**Figure S7. Proteomic analysis of lung tissues following treatment with Meis1-Exo@Cur.** (A) Schematic of the proteomic analysis workflow (Created in BioRender); (B) Volcano plot illustrating the distribution of DEPs; (C) Heatmap of protein expression levels from proteomic data; (D) Venn diagram showing the overlap between DEGs and DEPs; (E-F) Functional enrichment analysis of overlapping genes, including KEGG (E) and GO (F) analyses. Model: n = 3; Treatment: n = 3.

**Table S1. Sequence of RT-qPCR.**

| **Name** | **Sequence (5’-3’)** |
| --- | --- |
| Meis1 | F: CCATAGTCCCACCTTGGAGC |
|  | R: ATGCATGCCACCTTGCACTA |
| GAPDH | F: CCCTTAAGAGGGATGCTGCC |
|  | R: TACGGCCAAATCCGTTCACA |

Abbreviations: F, forward; R, reverse

**Table S2. Primary antibody information.**

| **Name** | **Cat.NO** | **Dilution ratio** | **Manufactor** |
| --- | --- | --- | --- |
| Meis1 | 67260 | 1: 1000 | CST |
| Cleaved Caspase-3 | ab214430 | 1: 5000 | Abcam |
| Bax | ab32503 | 1:5000 | Abcam |
| Bcl2 | ab182858 | 1:2000 | Abcam |
| CD9 | ab307085 | 1:1000 | Abcam |
| CD63 | ab315108 | 1:1000 | Abcam |
| CD81 | ab109201 | 1:1000 | Abcam |
| TSG101 | ab125011 | 1:1000 | Abcam |
| Calnexin | ab22595 | 1:1000 | Abcam |
| NF-κB (rabbit) | ab32536 | 1: 5000 | Abcam |
| p-NF-κB (rabbit) | ab76302 | 1: 1000 | Abcam |
| p38 MAPK (rabbit) | 9212 | 1: 1000 | CST |
| p-p38 MAPK (rabbit) | 9211 | 1: 1000 | CST |
| Nrf2 | 12721 | 1: 1000 | CST |
| HO-1 | ab189491 | 1: 2000 | Abcam |
| GAPDH | ab181602 | 1:10000 | Abcam |

**Table S3. Summary of Experimental Animal Usage**

| Experimental Group | Pre-set Sample Size  (n) | Actual Effective Sample Size  (n) | Purpose |
| --- | --- | --- | --- |
| Control Group | 6 | 6 | Baseline Control |
| Untreated Group | 6 | 6 | Injury Control |
| Exo Treatment Group | 6 | 6 | Efficacy Evaluation |
| Meis1-Exo Treatment Group | 6 | 6 | Efficacy Evaluation |
| Meis1-Exo@Cur Treatment Group | 6 | 6 | Efficacy Evaluation |
| Reserve/Supplementary | 6 | - | Contingency for modeling failure/attrition |
| *In Vivo* Biodistribution Study | 12 | 12 | Biodistribution Analysis |
| Total | 48 | 42 | - |

**Table S4. Antibodies Used for Flow Cytometric Analysis of Immune Cell Infiltration in Lung Tissue**

| Fluorophore | Target | Clone | Supplier | Catalog No. | Recommended Dosage  (per test) | Dilution Ratio |
| --- | --- | --- | --- | --- | --- | --- |
| APC/Cy7 | CD45 | 30-F11 | BioLegend | 103116 | 0.25 µg | 0.25 µg |
| FITC | CD11b | M1/70 | BioLegend | 101206 | 0.5 µg | 0.5 µg |
| PE/Cy7 | Ly6G | 1A8 | BioLegend | 127618 | 0.25 µg | 0.25 µg |
| PerCP/Cy5.5 | F4/80 | BM8 | BioLegend | 123128 | 0.25 µg | 0.25 µg |
| BV421 | CD86 | GL-1 | BioLegend | 105032 | 0.25 µg | 0.25 µg |
| APC | CD206 | C068C2 | BioLegend | 141708 | 0.5 µg | 0.5 µg |
| PE | CD3 | 17A2 | BioLegend | 100208 | 0.5 µg | 0.5 µg |
| BV605 | CD4 | GK1.5 | BioLegend | 100548 | 0.25 µg | 0.25 µg |
| BV785 | Foxp3 | MF-14 | BioLegend | 320426 | 0.25 µg | 0.25 µg |
| Purified | CD16/32 | 93 | BioLegend | 101302 | 0.5 µg (for blocking) | 0.5 µg |
